# FAM83H and SCRIB stabilize β-catenin and stimulate progression of gastric carcinoma

**DOI:** 10.18632/aging.103351

**Published:** 2020-06-20

**Authors:** Usama Khamis Hussein, Sang Hoon Ha, Asmaa Gamal Ahmed, Kyoung Min Kim, See-Hyoung Park, Chan Young Kim, Keun Sang Kwon, Zhongkai Zhang, Sang-A Lee, Ho Sung Park, Byung-Hyun Park, Ho Lee, Myoung Ja Chung, Woo Sung Moon, Myoung Jae Kang, Kyu Yun Jang

**Affiliations:** 1 Department of Pathology, Jeonbuk National University Medical School, Jeonju, Republic of Korea; 2 Research Institute of Clinical Medicine of Jeonbuk National University-Biomedical Research Institute of Jeonbuk National University Hospital, Jeonju, Republic of Korea; 3 Faculty of Science, Beni-Suef University, Beni-Suef, Egypt; 4 Division of Biotechnology, Jeonbuk National University, Iksan, Republic of Korea; 5 Faculty of Postgraduate Studies and Advanced Sciences, Beni-Suef University, Beni-Suef, Egypt; 6 Department of Bio and Chemical Engineering, Hongik University, Sejong, Republic of Korea; 7 Department of Surgery, Jeonbuk National University Medical School, Jeonju, Republic of Korea; 8 Department of Preventive Medicine, Jeonbuk National University Medical School, Jeonju, Republic of Korea; 9 Department of Biochemistry, Jeonbuk National University Medical School, Jeonju, Republic of Korea; 10 Department of Forensic Medicine, Jeonbuk National University Medical School, Jeonju, Republic of Korea

**Keywords:** stomach, cancer, FAM83H, SCRIB, β-catenin

## Abstract

FAM83H primarily is known for its function in tooth development. Recently, a role for FAM83H in tumorigenesis, conjunction with MYC and β-catenin, has been suggested. Analysis of public data indicates that FAM83H expression is closely associated with SCRIB expression in human gastric cancers. Therefore, this study investigated the roles of FAM83H and SCRIB in 200 human gastric cancers and gastric cancer cells. In human gastric carcinomas, both the individual and combined expression patterns of the nuclear FAM83H and SCRIB were independent indicators of shorter survival of gastric carcinoma patients. In MKN-45 and NCI-N87 gastric cancer cells, the expression of FAM83H and SCRIB were associated with proliferation and invasiveness of cells. FAM83H-mediated *in vivo* tumor growth was attenuated with knock-down of SCRIB. Moreover, immunoprecipitation indicates that FAM83H, SCRIB, and β-catenin, form a complex, and knock-down of either FAM83H or SCRIB accelerated proteasomal degradation of β-catenin. In conclusion, this study has found that the individual and combined expression patterns of nuclear FAM83H and SCRIB are prognostic indicators of gastric carcinomas and further suggests that FAM83H and SCRIB are involved in the progression of gastric carcinomas by stabilizing β-catenin.

## INTRODUCTION

FAM83H plays a pivotal role in dental enamel formation and in amelogenesis imperfecta [1, 2]. However, the effects of knocking out FAM83H in mice are not restricted to teeth development [3]. In particular, knock-out of FAM83H retarded overall growth of mice and induced malformation of digits, and abnormal teeth development was not part of the phenotype of all the knock-out mice [3]. These aberrant phenotypes in FAM83H knock-out mice suggest that FAM83H can have a pleiotropic role in cell proliferation and the molecules interacting with FAM83H might be involved in various roles of FAM83H [3]. In this aspect, the oncogene MYC is presented as a transcriptional regulator of FAM83H in hepatocellular carcinomas [4]. In kidney cancers, FAM83H stimulates cancer progression in cooperation with PANX2, and PANX2 has been suggested to be part of the down-stream signaling of FAM83H [5]. In addition, the cBioPortal database (http://www.cbioportal.org) provided evidence that FAM83H expression is closely associated with the expression of SCRIB in gastric cancers [6, 7]. Moreover, FAM83H is involved in various aspects of cell biology, including tumorigenesis. Higher expression of FAM83H increases the proliferation and invasiveness of cancer cells [4, 8–10]. The expression of FAM83H is higher in human cancer tissue compared with normal tissue [10–12] and higher expression of FAM83H is associated with poor prognosis of cancers of the uterus [10, 12], liver [4], kidney [5], and bone [8]. However, FAM83H is a favorable prognostic factor of glioma and head and neck cancers [12]. Therefore, tumorigenic roles of FAM83H might differ according to cancer types, or the molecule(s) interacting with FAM83H during tumorigenesis of specific cancer types.

SCRIB (scribble) is a polarity protein expressed on the basolateral side of epithelial cells and is important in maintaining tight junctions [13]. The loss of SCRIB in malignant tumors suggest that it has potential to be a tumor suppressor [14]. Alteration of polarity through deletion, downregulation, overexpression, and mislocalization can induce structural and functional alteration of cells that might be related to tumorigenesis [15, 16]. Mislocalization of SCRIB is involved in epithelial-to-mesenchymal transition (EMT) by inducing loss of E-cadherin [15, 17, 18]. Deregulated SCRIB expression induces tumorigenesis in breast [19] and liver [20].

Gastric cancer is ranked fifth in incidence [21] and third in cancer-associated death worldwide [21, 22]. The survival rate of gastric cancer is primarily associated with cancer stage and histological phenotype [22]. Especially, the survival rate of gastric cancer with high tumor stage and poorly cohesive carcinoma with fibrous stroma remains low [22]. Therefore, identification of histologic and molecular profiles in the progression of gastric cancer is important for the future treatment of gastric cancers. In this respect, FAM83H and SCRIB might be molecular therapeutic targets of gastric carcinomas. Based on the characteristic of FAM83H, SCRIB, and β-catenin as components of cell junctions [15], alteration of FAM83H, SCRIB, and β-catenin might stimulate the progression of gastric carcinomas. Moreover, FAM83H is important in the stabilization of β-catenin in osteosarcoma in our previous study [8], and a public database indicates a significant correlation between the expression of FAM83H and SCRIB in gastric cancer [6, 7]. The direct interaction of SCRIB with β-catenin has also been observed in synaptic vesicles [23]. Therefore, this study investigated the roles and associations between FAM83H, SCRIB, and β-catenin in gastric carcinomas using human gastric carcinoma tissues and gastric cancer cells.

## RESULTS

### The expression of FAM83H, SCRIB, and β-catenin in gastric carcinomas

**Figure 1:**
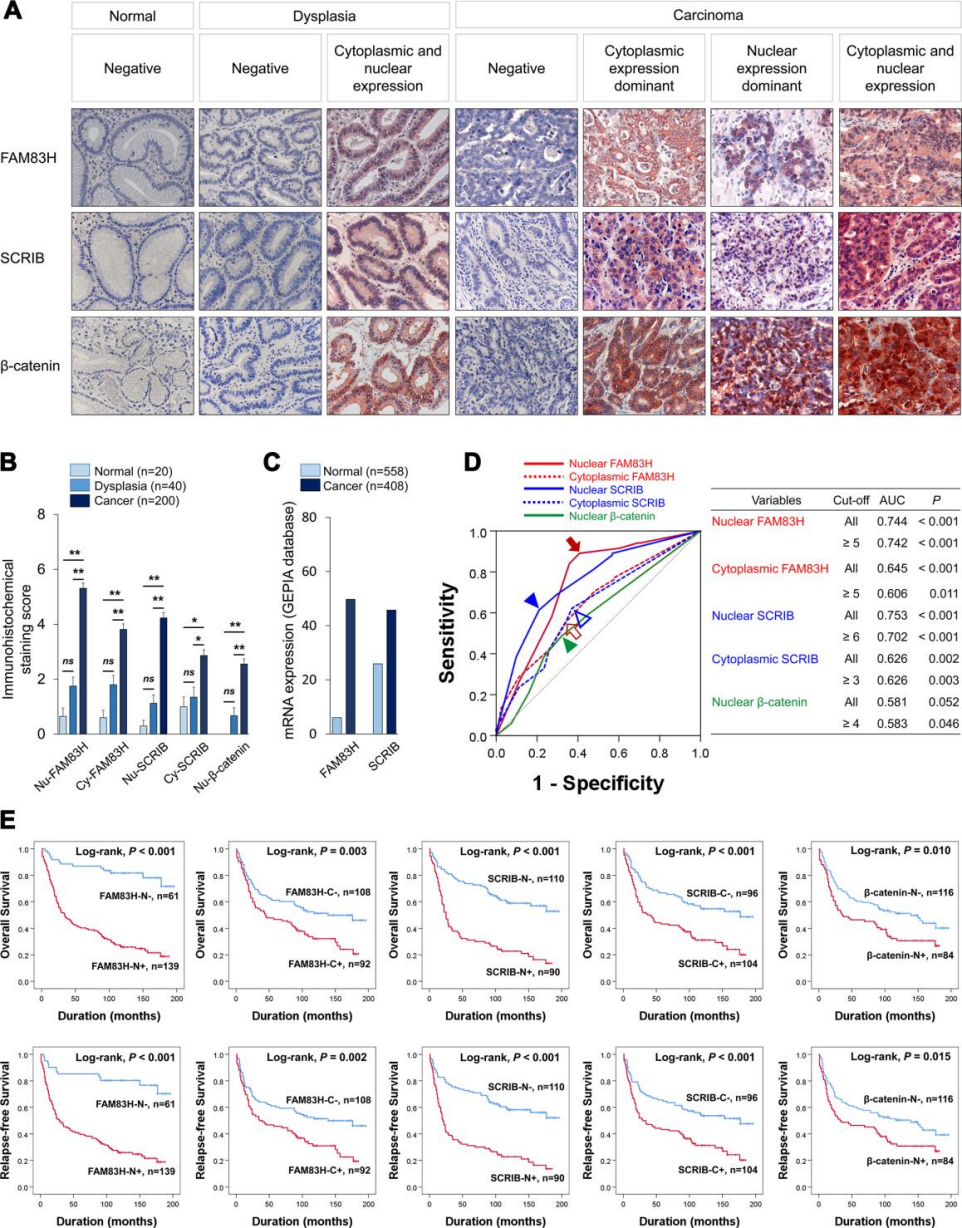
**Expression and prognostic significance of the expression of FAM83H, SCRIB, and β-catenin in 200 human gastric carcinomas.** (**A**) Immunohistochemical expression of FAM83H, SCRIB, and β-catenin in normal gastric mucosa, gastric dysplasia, and gastric carcinoma tissue. FAM83H and SCRIB are expressed in both the cytoplasm and nuclei of tumor cells. Original magnification: x400. (**B**) Immunohistochemical staining scores for nuclear FAM83H (FAM83H-N), cytoplasmic FAM83H (FAM83H-C), nuclear SCRIB (SCRIB-N), cytoplasmic SCRIB (SCRIB-C), and nuclear β-catenin (β-catenin-N) in 20 normal gastric mucosa, 40 gastric dysplasia, and 200 gastric carcinomas. (**C**) The expression of mRNA of FAM83H and SCRIB in normal gastric tissue and gastric cancers from the GEPIA database (http://gepia.cancer-pku.cn. Accessed 10 January 2020). (**D**) ROC curve analysis to determine cut-off points for the expression of FAM83H-N (red arrow), FAM83H-C (red empty arrow), SCRIB-N (blue arrowhead), SCRIB-C (blue empty arrowhead), and β-catenin-N (green arrowhead). The cut-off points are determined at the point of the highest area under the curve (AUC) to predict the death of gastric carcinoma patients. (**E**) Kaplan-Meier survival analysis of overall survival and relapse-free survival according to the FAM83H-N, FAM83H-C, SCRIB-N, SCRIB-C, and β-catenin-N expression.

In human tissue, both the nuclear and cytoplasmic expression were identified in FAM83H, SRCIB, and β-catenin immunostaining (Figure 1A). Immuno-histochemical staining scores for the expression of FAM83H in nuclei (FAM83H-N) and cytoplasm (FAM83H-H), expression of SCRIB in nuclei (SCRIB-N) and cytoplasm (SCRIB-C), and the expression of β-catenin in nuclei (β-catenin-N) were significantly higher in gastric cancers compared with normal gastric mucosa and gastric dysplasia (Figure 1B). Consistently, mRNA levels of FAM83H and SCRIB were higher in gastric cancers compared with normal gastric tissue in the GEPIA database (http://gepia.cancer-pku.cn. Accessed 10 January 2020) (Figure 1C). The cut-off points for FAM83H-N, FAM83H-C, SCRIB-N, SCRIB-C, and β-catenin-N expression were five, five, six, three, and four, respectively (Figure 1D). With these cut-off values, FAM83H-N was significantly associated with serum level of CA 19-9, tumor stage, tumor invasion, lymph node metastasis, venous invasion, histologic grade, and the expression of FAM83H-C, SCRIB-N, SCRIB-C, and β-catenin-N (Table 1). FAM83H-C was significantly associated with Lauren classification, and the expression of SCRIB-N, SCRIB-C, and β-catenin-N (Table 1). SCRIB-N was significantly associated with tumor stage, tumor invasion, lymph node metastasis, WHO classification of gastric cancer, and the expression of SCRIB-C and β-catenin-N (Table 1). SCRIB-C was significantly associated with age, WHO classification of gastric cancer, histologic grade, Lauren classification, and the expression of β-catenin-N (Table 1). Positive β-catenin-N expression was significantly associated with age, sex, and tumor stage (Table 1).

**Table 1:** Clinicopathologic variables and the expression of FAM83H, SCRIB, and β-catenin in gastric carcinomas.

**Characteristics**		**No.**	**FAM83H-N**			**FAM83H-C**			**SCRIB-N**			**SCRIB-C**			**β-catenin-N**	
			**Positive**	***P***		**Positive**	***P***		**Positive**	***P***		**Positive**	***P***		**Positive**	***P***
Age, years	< 60	59	37 (63%)	0.177		23 (39%)	0.198		24 (41%)	0.427		22 (37%)	0.007		16 (27%)	0.006
	≥ 60	141	102 (72%)			69 (49%)			66 (47%)			82 (58%)			68 (48%)	
Sex	Female	48	30 (63%)	0.227		18 (38%)	0.175		25 (52%)	0.258		28 (58%)	0.314		30 (63%)	< 0.001
	Male	152	109 (72%)			74 (49%)			65 (43%)			76 (50%)			54 (36%)	
CEA^*^	Normal	140	94 (67%)	0.079		64 (46%)	0.812		59 (42%)	0.650		69 (49%)	0.687		58 (41%)	0.848
	Elevated	30	25 (83%)			13 (43%)			14 (47%)			16 (53%)			13 (43%)	
CA19-9^*^	Normal	150	102 (68%)	0.043		68 (45%)	0.978		63 (42%)	0.497		74 (49%)	0.634		63 (42%)	0.865
	Elevated	20	18 (90%)			9 (45%)			10 (50%)			11 (55%)			8 (40%)	
TNM stage	I & II	99	52 (53%)	< 0.001		40 (40%)	0.116		34 (34%)	0.003		47 (47%)	0.205		34 (34%)	0.030
	III & IV	101	87 (86%)			52 (51%)			56 (55%)			57 (56%)			50 (50%)	
Tumor invasion	EGC	50	25 (50%)	< 0.001		19 (38%)	0.190		14 (28%)	0.005		21 (42%)	0.102		17 (34%)	0.186
	AGC	150	114 (76%)			73 (49%)			76 (51%)			83 (55%)			67 (45%)	
LN metastasis	Absence	77	42 (55%)	< 0.001		29 (38%)	0.061		25 (32%)	0.005		35 (45%)	0.143		27 (35%)	0.116
	Presence	123	97 (79%)			63 (51%)			65 (53%)			69 (56%)			57 (46%)	
Venous invasion	Absence	171	113 (66%)	0.011		81 (47%)	0.346		76 (44%)	0.701		88 (51%)	0.712		69 (40%)	0.251
	Presence	29	26 (90%)			11 (38%)			14 (48%)			16 (55%)			15 (52%)	
WHO classification	Tubular	138	100 (72%)	0.109		71 (51%)	0.141		71 (51%)	0.020		84 (61%)	< 0.001		60 (43%)	0.808
	PCC	20	10 (50%)			4 (20%)			4 (20%)			4 (20%)			6 (30%)	
	Mucinous	20	11 (55%)			9 (45%)			5 (25%)			6 (30%)			7 (35%)	
	Mixed	18	15 (83%)			6 (33%)			7 (39%)			6 (33%)			9 (50%)	
	Papillary	2	1 (50%)			1 (50%)			2 (100%)			2 (100%)			1 (50%)	
	Neuroendocrine	2	2 (100%)			1 (50%)			1 (50%)			2 (100%)			1 (50%)	
Histologic grade^**^	WD	13	5 (38%)	0.017		6 (46%)	0.413		3 (23%)	0.088		6 (46%)	0.015		3 (23%)	0.263
	MD	76	58 (76%)			43 (57%)			42 (55%)			55 (72%)			36 (47%)	
	PD	51	38 (75%)			23 (45%)			28 (55%)			25 (49%)			22 (43%)	
Lauren classification	Intestinal	96	67 (70%)	0.978		52 (54%)	0.036		46 (48%)	0.144		61 (64%)	< 0.001		43 (45%)	0.185
	Diffuse	80	55 (69%)			28 (35%)			30 (38%)			26 (33%)			28 (35%)	
	Mixed	24	17 (71%)			12 (50%)			14 (58%)			17 (71%)			13 (54%)	
β-catenin-N	Negative	116	68 (59%)	< 0.001		43 (27%)	0.003		36 (31%)	< 0.001		40 (34%)	< 0.001			
	Positive	84	71 (85%)			49 (58%)			54 (64%)			64 (76%)				
SCRIB-C	Negative	96	57 (59%)	0.003		23 (24%)	< 0.001		10 (10%)	< 0.001						
	Positive	104	82 (79%)			69 (66%)			80 (77%)							
SCRIB-N	Negative	110	60 (55%)	< 0.001		32 (29%)	< 0.001									
	Positive	90	79 (88%)			60 (67%)										
FAM83H-C	Negative	108	57 (53%)	< 0.001												
	Positive	92	82 (89%)													

Abbreviations: FAM83H-N, nuclear expression of FAM83H; FAM83H-C, cytoplasmic expression of FAM83H; SCRIB-N, nuclear expression of SCRIB; SCRIB-C, cytoplasmic expression of SCRIB; β-catenin-N, nuclear expression of β-catenin; CEA, carcinoembryonic antigen; CA19-9, carbohydrate antigen 19-9; LN, lymph node; WD, well-differentiated; MD, moderately differentiated; PD, poorly differentiated; PCC, poorly cohesive carcinoma; EGC, early gastric cancer; AGC, advanced gastric cancer; *; Preoperative serum level of CEA or CA19-9 were not measured in 30 patients. **; Histologic grading was carried in tubular and papillary type carcinomas according to the grading system of the WHO histological classification of gastric tumours.

### The expression of FAM83H, SCRIB, and β-catenin are associated with poor prognosis of gastric carcinoma patients with univariate analysis

**Figure 2:**
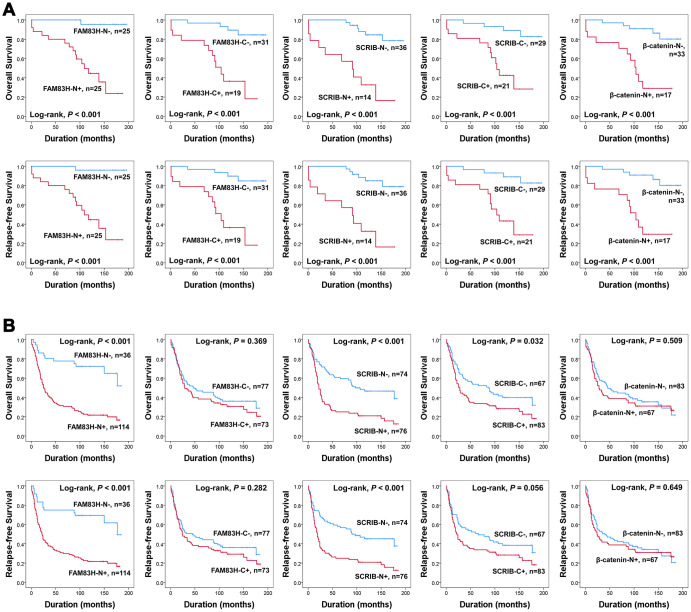
**Survival analysis according to the expression of FAM83H, SCRIB, and β-catenin in early gastric cancers and advanced gastric cancers.** Kaplan-Meier survival curves for overall survival and relapse-free survival in 50 early gastric cancers (**A**) and 150 advanced gastric cancers (**B**).

In univariate survival analysis, the factors significantly associated with both overall survival (OS) and relapse-free survival (RFS) were preoperative serum level of CEA (OS; *P* = 0.002, RFS; *P* = 0.002) and CA19-9 (OS; *P* = 0.002, RFS; *P* = 0.003), tumor stage (OS; *P* < 0.001, RFS; *P* < 0.001), lymph node metastasis (OS; *P* < 0.001, RFS; *P* < 0.001), venous invasion (OS; *P* < 0.001, RFS; *P* < 0.001), tumor invasion (OS; *P* < 0.001, RFS; *P* < 0.001), and the expression of FAM83H-N (OS; *P* < 0.001, RFS; *P* < 0.001), FAM83H-C (OS; *P* = 0.004, RFS; *P* = 0.002), SCRIB-N (OS; *P* < 0.001, RFS; *P* < 0.001), SCRIB-C (OS; *P* < 0.001, RFS; *P* < 0.001), and β-catenin-N (OS; *P* = 0.011, RFS; *P* = 0.017) (Table 2). The prognostic impacts of the expression of FAM83H-N, FAM83H-C, SCRIB-N, SCRIB-C, and β-catenin-N for OS and RFS are presented as Kaplan-Meier survival curves in Figure 1E. In further survival analysis in subgroups of early gastric cancers and advanced gastric cancers, the expression of FAM83H-N, FAM83H-C, SCRIB-N, SCRIB-C, and β-catenin-N were significantly associated with OS and RFS of early gastric carcinoma patients (Figure 2A). In 150 advanced gastric cancers, the expression of FAM83H-N and SCRIB-N were significantly associated with OS and RFS of gastric carcinoma patients (Figure 2B).

**Table 2:** Univariate Cox proportional hazards regression analysis for overall survival and relapse-free survival in gastric carcinoma patients.

**Characteristics**	**No.**	**OS**			**RFS**	
		**HR (95% CI)**	***P***		**HR (95% CI)**	***P***
Age, years, ≥ 60 (*vs* < 60)	141/200	1.295 (0.859-1.953)	0.217		1.309 (0.868-1.973)	0.198
Sex, male (*vs.* female)	152/200	0.936 (0.616-1.423)	0.936		0.954 (0.628-1.450)	0.954
CEA*, elevated (*vs.* normal)	30/170	2.084 (1.303-3.333)	0.002		2.092 (1.309-3.345)	0.002
CA19-9*, elevated (*vs.* normal)	20/170	2.344 (1.368-4.016)	0.002		2.240 (1.308-3.836)	0.003
TNM stage, III & IV (*vs.* I & II)	101/200	3.651 (2.463-5.412)	< 0.001		3.844 (2.592-5.699)	< 0.001
LN metastasis, presence (*vs.* absence)	150/200	2.393 (1.597-3.586)	< 0.001		2.488 (1.661-3.726)	< 0.001
Venous invasion, presence (*vs.* absence)	29/120	2.572 (1.636-4.043)	< 0.001		2.603 (1.656-4.090)	< 0.001
Tumor invasion, AGC (*vs.* EGC)	150/200	3.281 (1.933-5.570)	< 0.001		3.357 (1.978-5.698)	< 0.001
Histologic grade**, WD	13/140	1	0.084		1	0.094
MD	76/140	2.257 (0.898-5.675)	0.084		2.259 (0.899-5.681)	0.083
PD	51/140	2.832 (1.109-7.231)	0.029		2.782 (1.089-7.103)	0.032
FAM83H-N, positive (*vs.* negative)	139/200	5.943 (3.328-10.612)	< 0.001		5.407 (3.086-9.476)	< 0.001
FAM83H-C, positive (*vs.* negative)	92/200	1.713 (1.192-2.461)	0.004		1.756 (1.223-2.519)	0.002
SCRIB-N, positive (*vs.* negative)	90/200	3.094 (2.128-4.499)	< 0.001		2.972 (2.050-4.309)	< 0.001
SCRIB-C, positive (*vs.* negative)	104/200	2.053 (1.414-2.983)	< 0.001		1.972 (1.361-2.856)	< 0.001
β-catenin-N, positive (*vs.* negative)	84/200	1.597 (1.114-2.290)	0.011		1.550 (1.083-2.219)	0.017
FAM83H-N/SCRIB-N, -/- or -/+	61/200	1	< 0.001		1	< 0.001
+/-	60/200	3.947 (2.904-7.440)	< 0.001		3.617 (1.952-6.702)	< 0.001
+/+	79/200	8.400 (4.612-15.301)	< 0.001		7.563 (4.229-13.524)	< 0.001

Abbreviations: OS, overall survival; RFS, relapse-free survival; HR, hazard ratio; CEA, carcinoembryonic antigen; CA19-9, carbohydrate antigen 19-9; LN, lymph node; EGC, early gastric cancer; AGC, advanced gastric cancer; WD, well-differentiated; MD, moderately differentiated; PD, poorly differentiated; FAM83H-N, nuclear expression of FAM83H; FAM83H-C, cytoplasmic expression of FAM83H; SCRIB-N, nuclear expression of SCRIB; SCRIB-C, cytoplasmic expression of SCRIB; β-catenin-N, nuclear expression of β-catenin. *; Preoperative serum level of CEA or CA19-9 were not measured in 30 patients. **; Histologic grading was carried in tubular and papillary type carcinomas according to the grading system of the WHO histological classification of gastric tumours.

### Co-expression patterns of FAM83H-N and SCRIB-N were associated with poor prognosis of gastric carcinoma patients with univariate analysis

**Figure 3:**
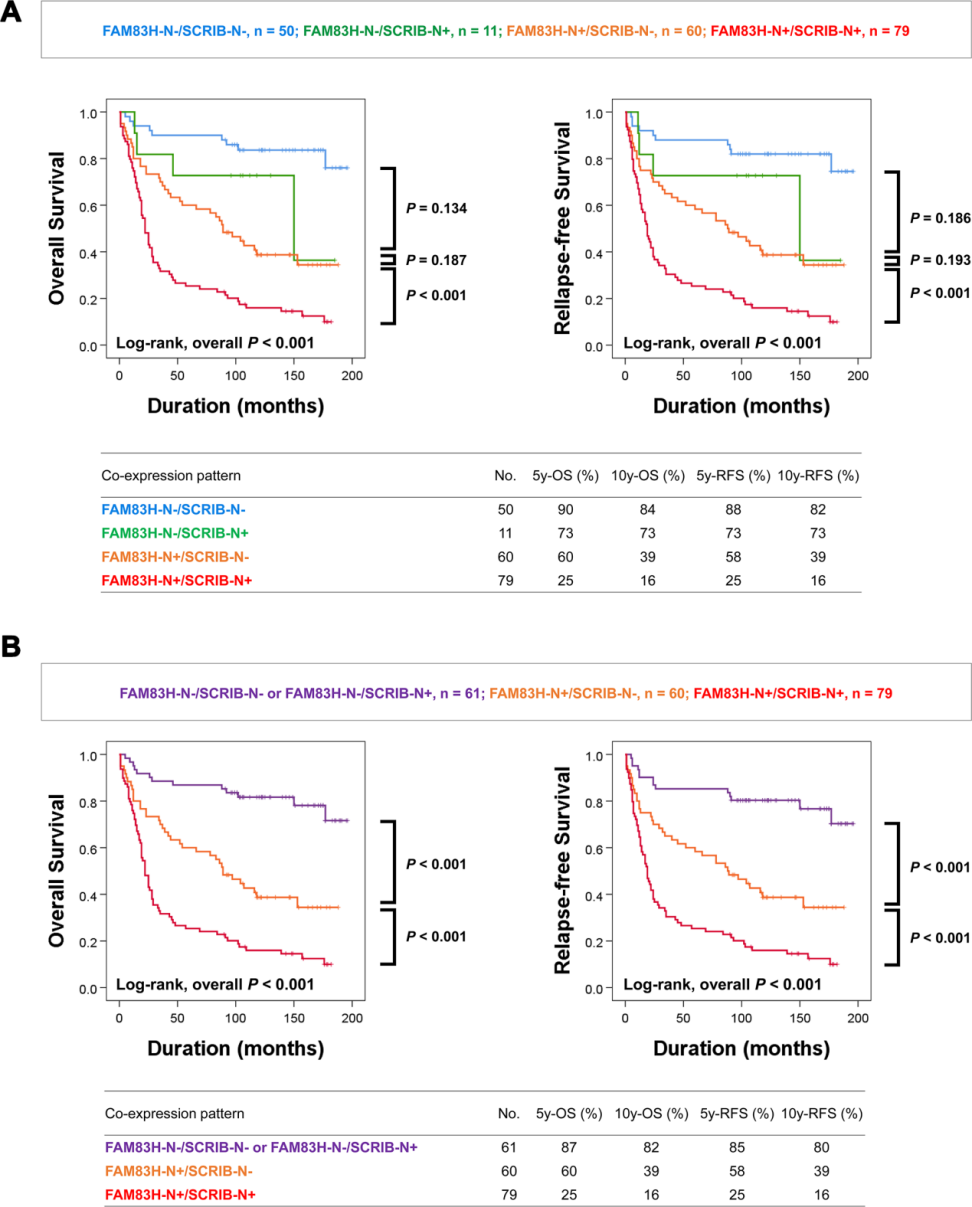
**Kaplan-Meier survival analysis according to co-expression patterns of nuclear FAM83H and nuclear SCRIB in gastric carcinoma patients.** (**A**) Kaplan-Meier survival curves for overall survival and relapse-free survival in four subgroups of gastric carcinomas: FAM83H-N^-^/SCRIB-N^-^, FAM83H-N^-^/SCRIB-N^+^, FAM83H-N^+^/SCRIB-N^-^, and FAM83H-N^+^/SCRIB-N^+^ subgroups. (**B**) Kaplan-Meier survival curves for overall survival and relapse-free survival in three of gastric carcinomas: FAM83H^-^/SCRIB-N^-^ or FAM83H-N^-^/SCRIB-N^+^, FAM83H-N^+^/SCRIB-N^-^, and FAM83H-N^+^/SCRIB-N^+^ subgroups. 5y-OS; overall survival rate at five years, 10y-OS; overall survival rate at ten years, 5y-RFS; relapse-free survival rate at five years, 10y-RFS; relapse-free survival rate at ten years.

In addition, as shown in Tables 1 and 2, there was a significant association between the expression of FAM83H-N, FAM83H-C, SCRIB-N, and SCRIB-C, and the prognostic impacts of FAM83H-N and SCRIB-N were stronger than FAM83H-C and SCRIB-C; the hazard ratios are greater in FAM83H-N and SCRIB-N expression compared with FAM83H-C and SCRIB-C expression. Based on these results, we further evaluated the prognostic impact of the co-expression patterns of FAM83H-N and SCRIB-N. When we sub-grouped gastric carcinomas into FAM83H-N^-^/SCRIB-N^-^, FAM83H-N^-^/SCRIB-N^+^, FAM83H-N^+^/SCRIB-N^-^, and FAM83H-N^+^/SCRIB-N^+^ subgroups, the FAM83H-N^-^/SCRIB-N^-^ subgroup had the longest survival and the FAM83H-N^+^/SCRIB-N^+^ subgroup had the shortest survival (Figure 3A). However, the differences in survival between the FAM83H-N^-^/SCRIB-N^-^, FAM83H-N^-^/SCRIB-N^+^, and FAM83H-N^+^/SCRIB-N^-^ subgroups were not significant. Therefore, we re-grouped gastric carcinomas into three subgroups: favorable (FAM83H-N^-^/SCRIB-N^-^ or FAM83H-N^-^/SCRIB-N^+^), intermediate (FAM83H-N^+^/SCRIB-N^-^), and poor (FAM83H-N^+^/SCRIB-N^+^) subgroups. In-between these subgroups, there was a significant difference in OS and RFS. The 5- and 10-year OS rates of the favorable (FAM83H-N^-^/SCRIB-N^-^ or FAM83H-N^-^/SCRIB-N^+^) subgroup were 87% and 82%, respectively. The 5- and 10-year OS rates of the poor subgroup with FAM83H-N^+^ and SCRIB-N^+^ were 25% and 16%, respectively. The 5- and 10-year OS rates of the intermediate (FAM83H-N^+^/SCRIB-N^-^) subgroup were 60% and 39%, respectively (Figure 3B).

### Individual and co-expression patterns of FAM83H-N and SCRIB-N predict shorter survival of gastric carcinoma patients with multivariate analysis

Next, we performed multivariate analysis with the factors significantly associated with OS and RFS. Preoperative serum levels of CEA and CA19-9 were not included in multivariate analysis since the data were missing for 30 patients. In multivariate analysis, tumor stage, FAM83H-N positivity, and SCRIB-N positivity were independent indicators of poor prognosis for both OS and RFS of gastric carcinoma patients (Table 3, Multivariate analysis model 1). FAM83H-N positivity predicted a 3.551-fold greater risk of death and a 3.155-fold greater risk of relapse or death of patients. SCRIB-N positivity predicted a 2.128-fold greater risk of death and a 2.062-fold greater risk of relapse or death of patients. The co-expression pattern of FAM83H-N and SCRIB-N is also an independent indicator of poor prognosis of survival of gastric carcinoma patients (Table 3, multivariate analysis model 2). The FAM83H-N^+^/SCRIB-N^+^ poor prognostic subgroup had a 6.266-fold greater risk of death and a 5.442-fold greater risk of relapse or death of patients compared with the favorable prognostic subgroups (FAM83H-N^-^/SCRIB-N^-^ or FAM83H-N^-^/SCRIB-N^+^) (Table 3).

**Table 3:** Multivariate Cox regression analysis for overall survival and relapse-free survival.

**Characteristics**	**OS**			**RFS**	
	**HR (95% CI)**	***P***		**HR (95% CI)**	***P***
Multivariate analysis model 1*					
TNM stage, III & IV (*vs.* I & II)	2.536 (1.694-3.797)	< 0.001		2.681 (1.789-4.017)	< 0.001
FAM83H-N, positive (*vs.* negative)	3.551 (1.941-6.499)	< 0.001		3.155 (1.753-5.676)	< 0.001
SCRIB-N, positive (*vs.* negative)	2.128 (1.450-3.124)	< 0.001		2.062 (1.409-3.019)	< 0.001
Multivariate analysis model 2**					
TNM stage, III & IV (*vs.* I & II)	2.574 (1.717-3.859)	< 0.001		2.728 (1.817-4.096)	< 0.001
FAM83H-N/SCRIB-N, -/- or -/+	1	< 0.001		1	< 0.001
+/-	2.936 (1.536-5.611)	0.001		2.596 (1.378-4.888)	0.003
+/+	6.266 (3.384-11.600)	< 0.001		5.442 (2.988-9.914)	< 0.001

Abbreviations: OS, overall survival; RFS, relapse-free survival. In multivariate analysis, the factors significantly associated with OS and RFS were included in multivariate analysis. However, because CEA and CA19-9 were not measured in 30 patients, they were not included in multivariate analysis. *; Variables considered in the multivariate analysis model 1 were tumor stage, lymph node metastasis, venous invasion, tumor invasion (EGC versus AGC), nuclear expression of FAM83H, cytoplasmic expression of FAM83H, nuclear expression of SCRIB, cytoplasmic expression of SCRIB, and nuclear expression of β-catenin. **; Variables considered in the multivariate analysis model 2 were tumor stage, lymph node metastasis, venous invasion, tumor invasion (EGC versus AGC), nuclear expression of β-catenin, and co-expression patterns of nuclear FAM83H and nuclear SCRIB.

### FAM83H and SCRIB stimulate the proliferation and invasiveness of gastric cancer cells

**Figure 4:**
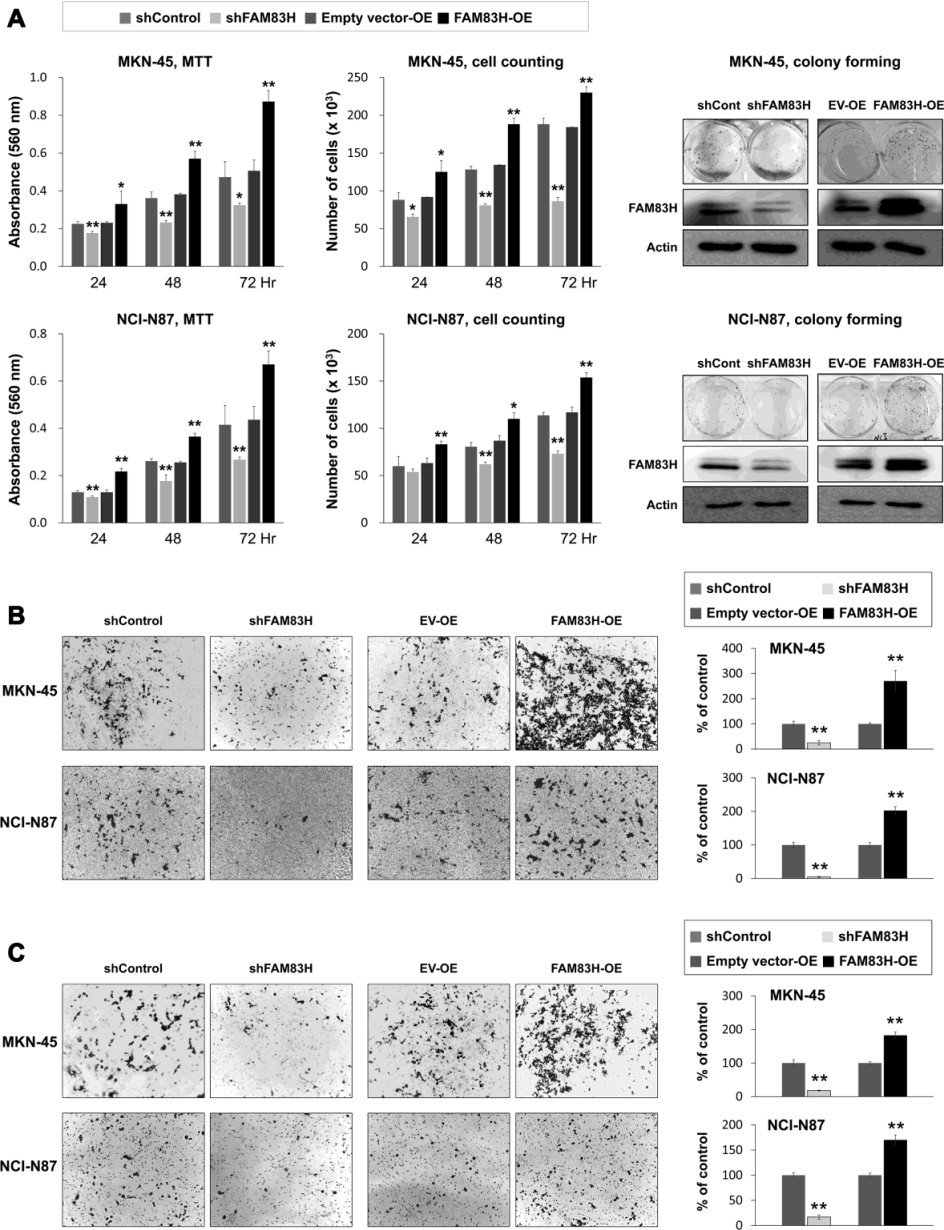
**The effect of knock-down or overexpression of FAM83H on the proliferation and invasiveness of gastric cancer cells.** (**A**) The effect of knock-down or overexpression of FAM83H on proliferation were evaluated with an MTT assay, cell counting, and a colony-forming assay in MKN-45 and NCI-N87 cells. The MTT assay was performed after seeding 3,000 cells per well of a 96-well plate. Cell counting was performed after seeding 3,000 cells per well of a 6-well plate. The colony-forming assay was performed by plating 1,000 cells per well in a 6-well plate for ten days. The knock-down or overexpression of FAM83H was assessed *via* western blot for FAM83H. (**B**) The migration assay was performed by seeding 1x10^5^ MKN-45 or 1x10^5^ NCI-N87 cells in the upper chamber for 48 hours. (**C**) The invasion assay was performed by seeding 2x10^5^ MKN-45 or 2x10^5^ NCI-N87 cells in the upper chamber with Matrigel for 48 hours. The chambers were stained with DIFF-Quik staining solution, and the number of migrated or invaded cells were counted in five x200 microscopic fields in each well. *; *P* < 0.05, **; *P* < 0.001.

**Figure 5:**
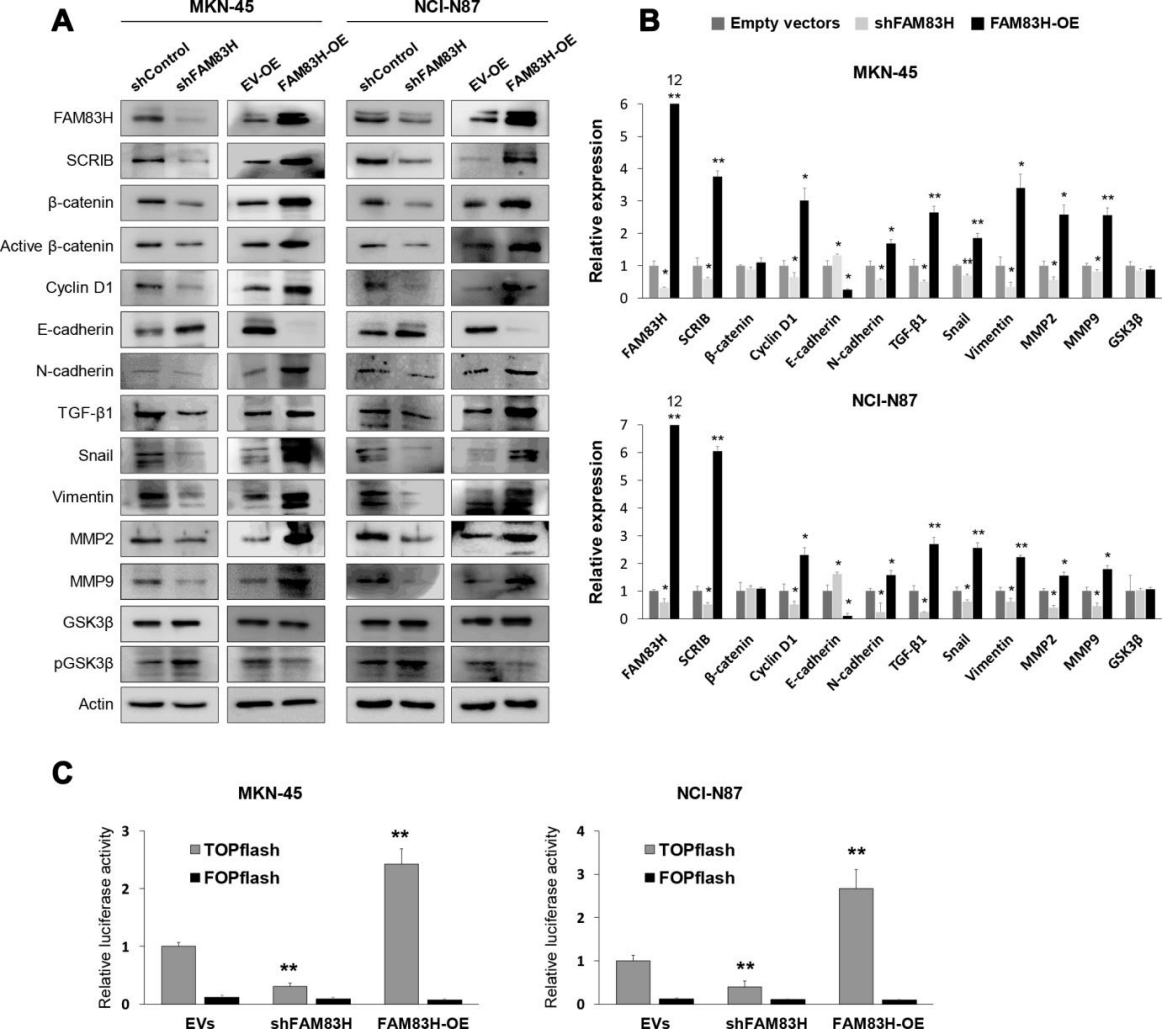
**Western blot, quantitative reverse-transcription polymerase chain reaction, and luciferase reporter assay after knock-down or overexpression of FAM83H in gastric cancer cells.** (**A**) Western blot was performed for FAM83H, SCRIB, β-catenin, active β-catenin, cyclin D1, E-cadherin, N-cadherin, TGF-β1, snail, vimentin, MMP2, MMP9, GSK3β, phosphorylated GSK3β, and actin after knock-down or overexpression of FAM83H in MKN-45 and NCI-N87 gastric cancer cells. (**B**) Quantitative reverse-transcription polymerase chain reaction was performed for FAM83H, SCRIB, β-catenin, cyclin D1, E-cadherin, N-cadherin, TGF-β1, snail, vimentin, MMP2, MMP9, and GSK3β after knock-down or overexpression of FAM83H in MKN-45 and NCI-N87 gastric cancer cells. (**C**) TOPflash luciferase reporter assay was performed by transfecting TOPflash or FOPflash plasmid DNA with pRL-TK *Renilla* Luciferase plasmid DNA in MKN-45 and NCI-N87 cells with induction of knock-down or overexpression of FAM83H. *; *P* < 0.05, **; *P* < 0.001.

Based on the prognostic significance of the expression of FAM83H and SCRIB in gastric carcinoma patients, we performed proliferation and migration/invasion assays after inducing knock-down or overexpression of FAM83H and SCRIB in gastric cancer cells. In MKN-45 and NCI-N87 gastric cancer cells, the proliferation of cells was inhibited with knock-down of FAM83H and increased with overexpression of FAM83H (Figure 4A). In addition, FAM83H stimulated the migration and invasion activity of MKN-45 and NCI-N87 cells (Figure 4B, 4C). Knock-down of FAM83H decreased protein and mRNA expression of SCRIB, cyclin D1, N-cadherin, TGF-β1, snail, vimentin, MMP2, and MMP9, and increased the expression of E-cadherin in both MKN-45 and NCI-N87 cells (Figure 5A, 5B). Overexpression of FAM83H increased protein and mRNA expression of SCRIB, cyclin D1, N-cadherin, TGF-β1, snail, vimentin, MMP2, and MMP9, and decreased the expression of E-cadherin in both MKN-45 and NCI-N87 cells (Figure 5A, 5B). Also, the knock-down of FAM83H decreased the protein levels of β-catenin and active β-catenin and overexpression of FAM83H increased the protein levels of β-catenin and active β-catenin. However, knock-down or overexpression of FAM83H did not affect mRNA expression of β-catenin (Figure 5A, 5B). The protein and mRNA expression of GSK3β were not altered with knock-down or overexpression of FAM83H (Figure 5A, 5B). However, knock-down of FAM83H increased the protein level of phosphorylated GSK3β, and overexpression of FAM83H decreased the protein level of phosphorylated GSK3β (Figure 5A). Furthermore, TOPflash reporter activity, but not FOPflash activity, was significantly decreased with knock-down of FAM83H and was significantly increased with overexpression of FAM83H (Figure 5C).

**Figure 6:**
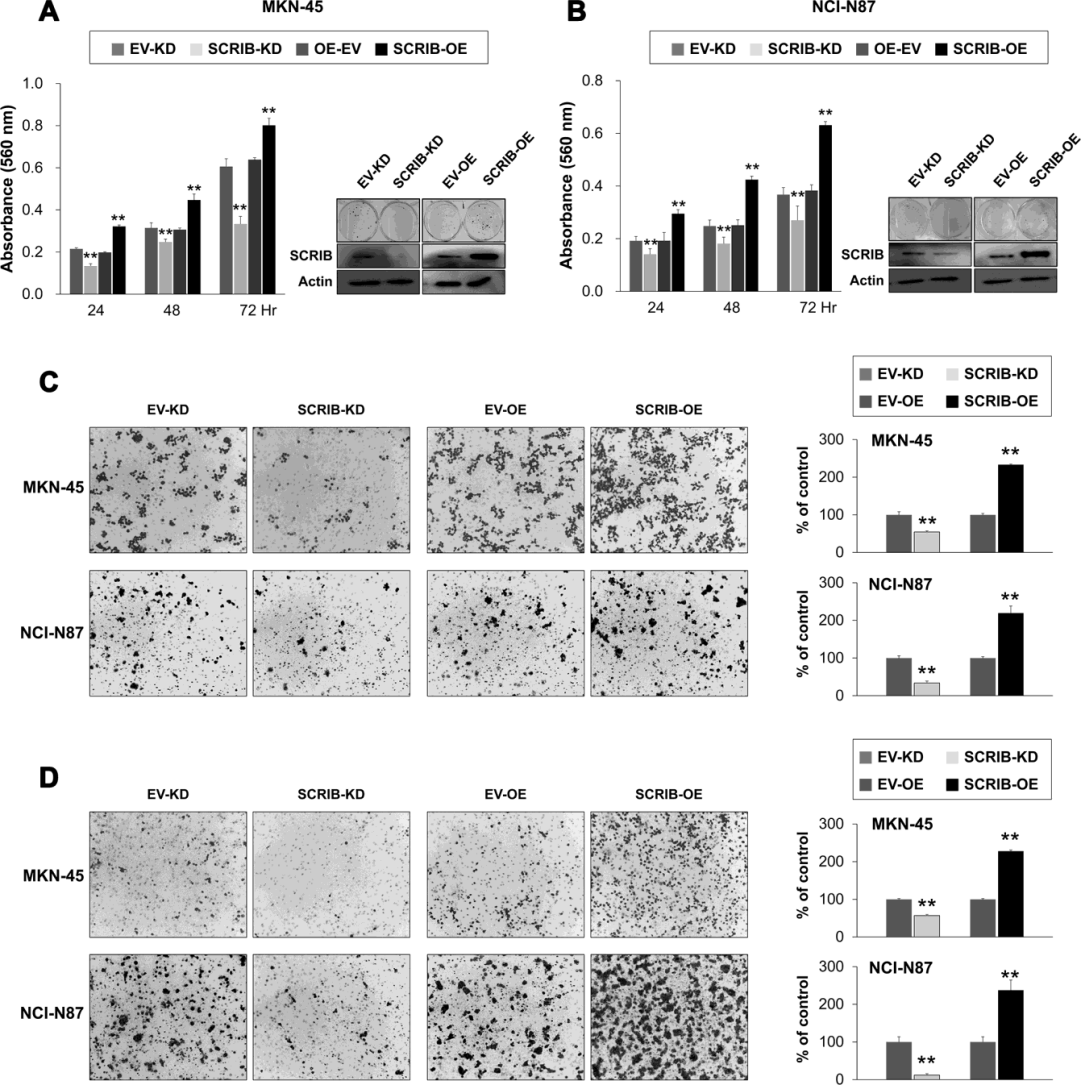
**The effect of knock-down or overexpression of SCRIB on the proliferation and invasiveness of gastric cancer cells.** (**A**, **B**) The effect of knock-down or overexpression of SCRIB on proliferation was evaluated with an MTT assay and colony-forming assay in MKN-45 (**A**) and NCI-N87 cells (**B**). The MTT assay was performed after seeding 3,000 cells per well of a 96-well plate. The colony-forming assay was performed by plating 1,000 cells per well in a 6-well plate for ten days. The knock-down or overexpression of SCRIB was assessed *via* western blot for SCRIB. (**C**) The migration assay was performed by seeding 1x10^5^ MKN-45 or 1x10^5^ NCI-N87 cells in the upper chamber for 48 hours. (**D**) The invasion assay was performed by seeding 2x10^5^ MKN-45 or 2x10^5^ NCI-N87 cells in the upper chamber with Matrigel for 48 hours. The chambers were stained with DIFF-Quik staining solution, and the number of migrated or invaded cells were counted in five x200 microscopic fields in each well. **; *P* < 0.001.

**Figure 7:**
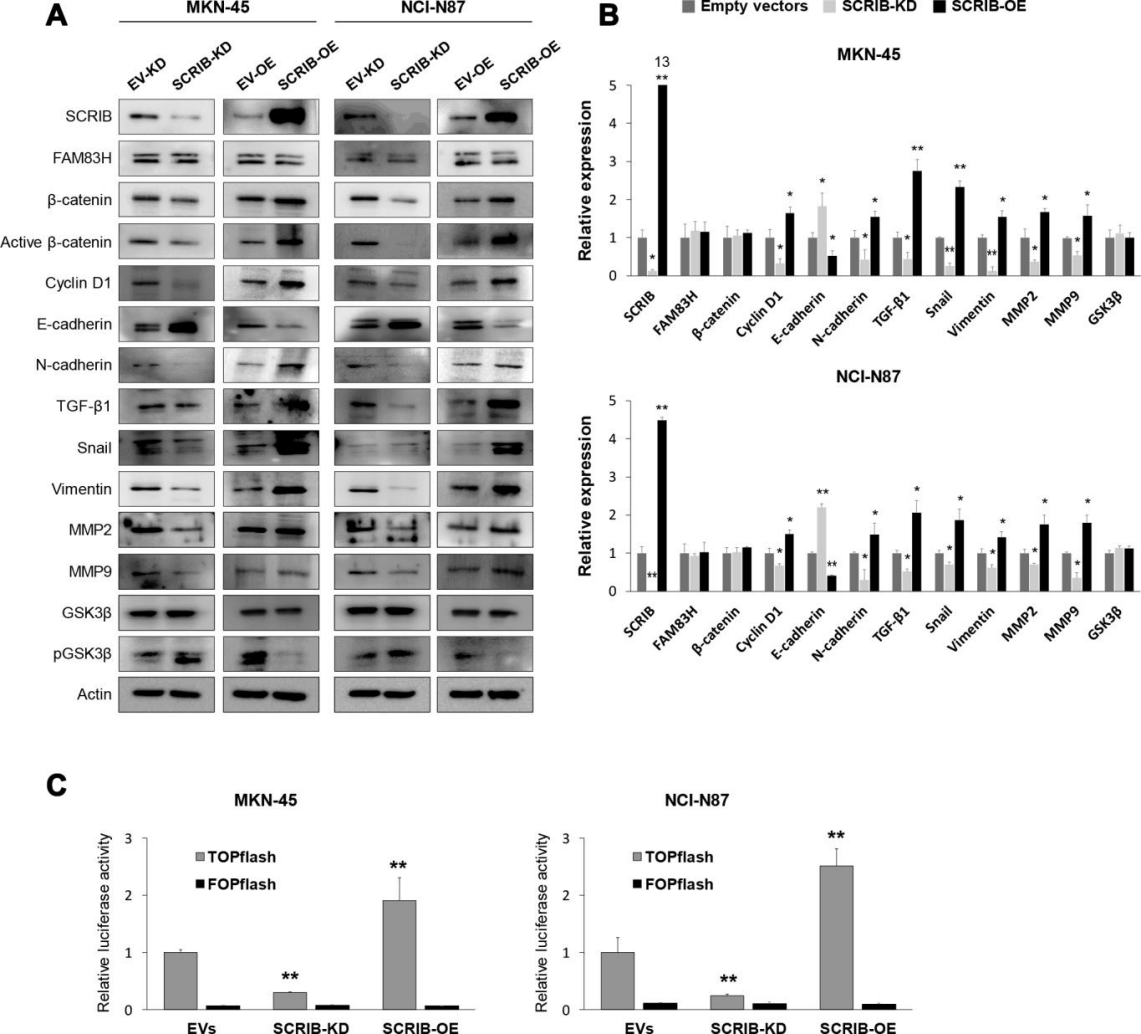
**Western blot and quantitative reverse-transcription polymerase chain reaction after knock-down or overexpression of SCRIB in gastric cancer cells.** (**A**) Western blot was performed for SCRIB, FAM83H, β-catenin, active β-catenin, cyclin D1, E-cadherin, N-cadherin, TGF-β1, snail, vimentin, MMP2, MMP9, GSK3β, phosphorylated GSK3β, and actin after knock-down or overexpression of SCRIB in MKN-45 and NCI-N87 gastric cancer cells. (**B**) Quantitative reverse-transcription polymerase chain reaction was performed for SCRIB, FAM83H, β-catenin, cyclin D1, E-cadherin, N-cadherin, TGF-β1, snail, vimentin, MMP2, MMP9, and GSK3β after knock-down or overexpression of SCRIB in MKN-45 and NCI-N87 gastric cancer cells. (**C**) TOPflash luciferase reporter assay was performed by transfecting TOPflash or FOPflash plasmid DNA with pRL-TK *Renilla* Luciferase plasmid DNA in MKN-45 and NCI-N87 cells with induction of knock-down or overexpression of SCRIB. *; *P* < 0.05, **; *P* < 0.001.

SCRIB also stimulated the proliferation and migration/invasion activity of MKN-45 and NCI-N87 gastric cancer cells (Figure 6A–6D). The proliferation and migration/invasion activity of cells were inhibited with knock-down of SCRIB and were increased with overexpression of SCRIB (Figure 6A–6D). Knock-down of SCRIB decreased the protein and mRNA expression of cyclin D1, N-cadherin, TGF-β1, snail, vimentin, MMP2, and MMP9, and increased the expression of E-cadherin (Figure 7A, 7B). Overexpression of SCRIB increased protein and mRNA expression of cyclin D1, N-cadherin, TGF-β1, snail, vimentin, MMP2, and MMP9, and decreased the expression of E-cadherin (Figure 7A, 7B). However, SCRIB did not affect the expression of FAM83H protein and mRNA (Figure 7A, 7B). Furthermore, the loss of SCRIB was associated with the decrease of the protein expression of β-catenin and active β-catenin. However, the mRNA expression of β-catenin was not decreased with the loss of SCRIB (Figure 7A, 7B). The protein and mRNA expression of GSK3β were not altered with knock-down or overexpression of SCRIB (Figure 7A, 7B). However, the phosphorylated GSK3β protein was increased with knock-down of SCRIB and decreased with overexpression of SCRIB (Figure 7A). Furthermore, TOPflash reporter activity, but not FOPflash activity, was significantly decreased with loss of SCRIB and was significantly increased with overexpression of SCRIB (Figure 7C).

### SCRIB mediates FAM83H-associated proliferation of gastric cancer cells

**Figure 8:**
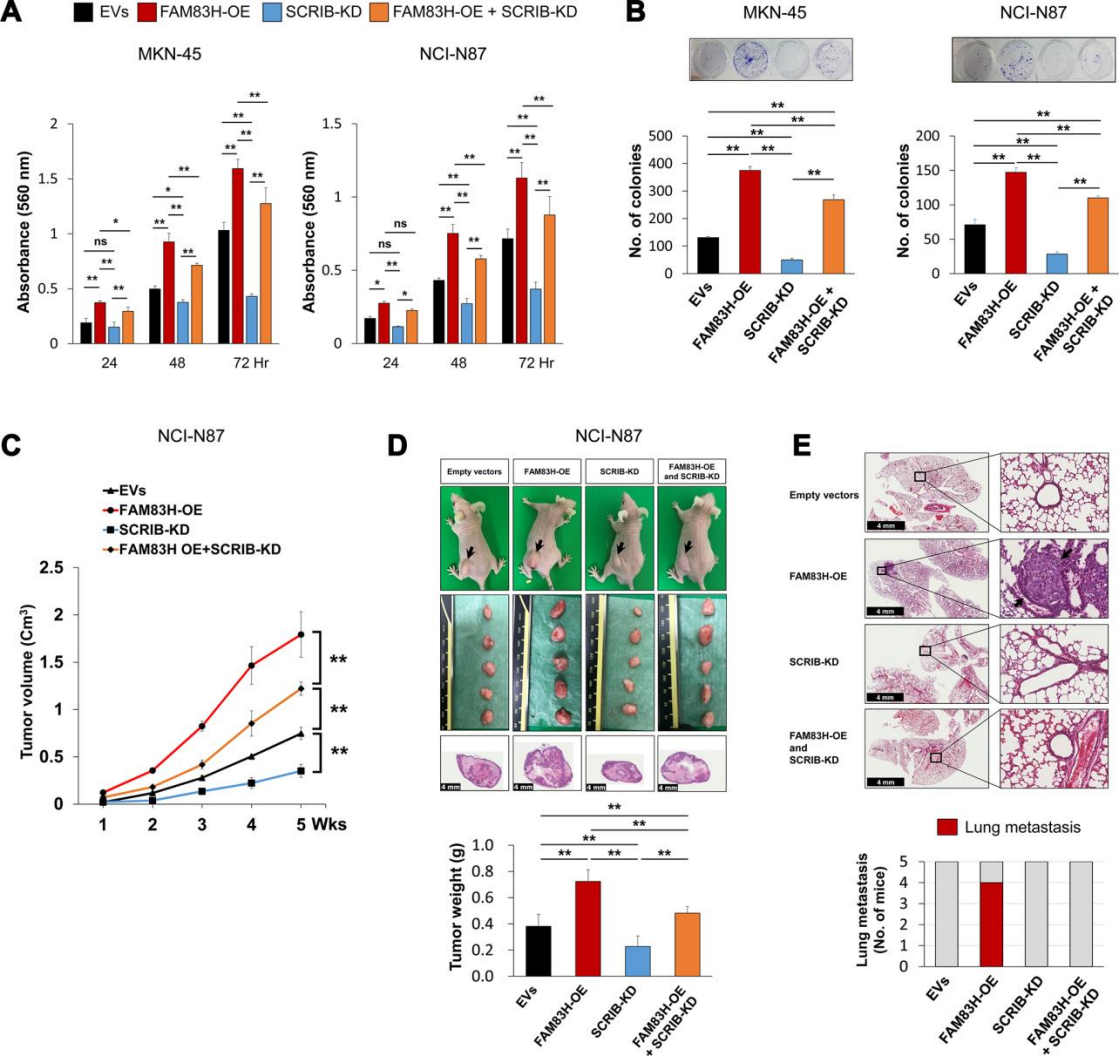
**The combined effect of overexpression of FAM83H and knock-down of SCRIB on the proliferation and tumor formation of gastric cancer cells.** (**A**, **B**) The effect of overexpression of FAM83H and/or knock-down of SCRIB on the proliferation of MKN45 and NCI-N87 gastric cancer cells were evaluated with an MTT assay (**A**) and a colony-forming assay (**B**). The MTT assay was performed after seeding 3,000 cells per well of a 96-well plate. The colony-forming assay was performed by plating 1,000 cells per well in a 6-well plate for ten days. The number of colonies was determined with GeneTools analysis software. (**C**, **D**) *In vivo* tumor growth was evaluated by subcutaneously implanting 2x10^6^ NCI-N87 cells with overexpression of FAM83H and/or knock-down of SCRIB. The tumor volume was measured every week after tumor implantation by the equation V = LxWxHx0.52 mm^3^ (**C**). At five weeks after tumor inoculation, the mice were euthanized and tumor weight was measured (**D**). (**E**) The mice were evaluated for metastasis and histologic findings of the lung. The arrows indicate metastatic NCI-N87 cells in lung. *; *P* < 0.05, **; *P* < 0.001.

FAM83H and SCRIB stimulate the proliferation and invasiveness of gastric cancer cells, and the expression of SCRIB was dependent on FAM83H expression, but FAM83H was not affected by SCRIB expression. Therefore, we evaluated the effects of knock-down of SCRIB on gastric cancer cells on inducing the overexpression of FAM83H. Consistent with the results in Figure 3, overexpression of FAM83H increased proliferation, which was attenuated with knock-down of SCRIB in MKN-45 and NCI-N87 cells (Figure 8A, 8B). Consistent with these findings, overexpression of FAM83H stimulated *in vivo* growth of NCI-N87 cells and loss of SCRIB suppressed *in vivo* tumor growth compared with controls transfected with empty vectors (Figure 8C, 8D). The *in vivo* tumor growth of the cells that were induced to overexpress FAM83H and have a knock-down of SCRIB was intermediate to those of the control and the FAM83H-overexpressing groups (Figure 8C, 8D). Moreover, pulmonary metastasis of gastric cancer cells was seen in four of five mice implanted NCI-N87 cells overexpressing FAM83H (Figure 8E). There was no pulmonary metastasis in mice implanted with NCI-N87 cells transfected with empty vectors, hSCRIB CRISPR/Cas9 KO vectors, or both the FAM83H overexpression vector and the hSCRIB CRISPR/Cas9 KO vectors (Figure 8E).

### FAM83H and SCRIB stabilize β-catenin by protecting it from proteasomal degradation

**Figure 9:**
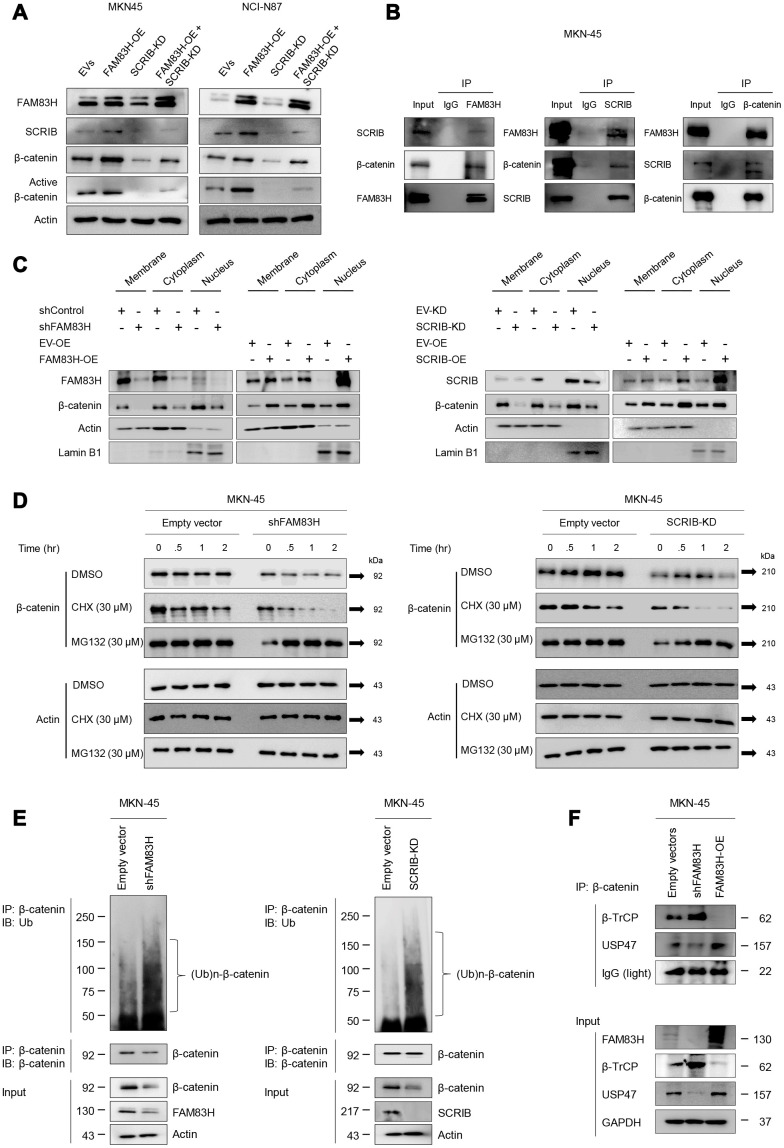
**FAM83H and SCRIB are involved in the stabilization of β-catenin from proteasomal ubiquitin degradation.** (**A**) Western blot for β-catenin and active β-catenin after overexpression of FAM83H and/or knock-down of SCRIB. (**B**) The protein lysate obtained from MKN-45 cells after immunoprecipitation with FAM83, SCRIB, or β-catenin. Thereafter, the immunoprecipitated protein was immunoblotted for FAM83H, SCRIB, and β-catenin. (**C**) Western blot for FAM83H, SCRIB, β-catenin, actin, and lamin B1 with the cytoplasmic membrane, cytoplasm, and nuclear protein lysates fractionated according to subcellular localization after inducing knock-down or overexpression of FAM83H or SCRIB in MKN-45 cells. (**D**) The MKN-45 cells were transfected with empty vector, shRNA for FAM83H, or hSCRIB CRISPR/Cas9 KO plasmid and treated with 30 μM cycloheximide or 30 μM MG132 for 0.5 to 4.0 hours. Thereafter, the total protein was immunoblotted for β-catenin and actin. (**E**) The MKN-45 cells were transfected with empty vector, shRNA for FAM83H, or hSCRIB CRISPR/Cas9 KO plasmid, and were treated with 30 μM MG132 for two hours. The protein lysate was immunoprecipitated with anti-β-catenin antibodies and immunoblotted with anti-ubiquitin antibodies. The immunoblot was performed on total protein lysate. (**F**) The MKN-45 cells were transfected with empty vectors, shRNA for FAM83H, or an overexpression vector for FAM83H. The protein lysate was immunoprecipitated with anti-β-catenin antibodies and immunoblotted with anti-β-TrCP, anti-USP47, or anti-GAPDH antibodies. The immunoblots were performed on total protein lysate.

Activation of the FAM83H-SCRIB pathway stimulates the proliferation and invasion of gastric cancer cells, and both FAM83H and SCRIB are involved in the β-catenin-related pathway and the EMT pathway. Interestingly, the expression of FAM83H and SCRIB influenced the protein levels of β-catenin and active β-catenin, but not the mRNA level of β-catenin. Co-transfection to induce the overexpression of FAM83H and knock-down of SCRIB affected the protein expression of β-catenin and active β-catenin (Figure 9A). Higher expression levels of β-catenin and active β-catenin protein *via* overexpression of FAM83H were attenuated with knock-down of SCRIB (Figure 9A). Moreover, western blotting of immunoprecipitates indicated that FAM83H, SCRIB, and β-catenin form a complex (Figure 9B). Furthermore, the nuclear fraction of β-catenin decreased with loss of FAM83H or SCRIB and increased with overexpression of FAM83H or SCRIB in cellular fractionation analysis of MKN-45 cells (Figure 9C). Therefore, to further explore the roles of FAM83H and SCRIB in the stabilization of β-catenin, we evaluated the proteasomal degradation of β-catenin. The β-catenin protein was rapidly degraded with the treatment of 30 μM cycloheximide in MKN-45 cells that had a knock-down of FAM83H or SCRIB (Figure 9D). Moreover, the ubiquitination of β-catenin was increased with the loss of FAM83H or SCRIB (Figure 9E). In the immunoprecipitated of β-catenin after knock-down of FAM83H or SCRIB, there was more poly-ubiquitinated β-catenin compared with controls (Figure 9E). Furthermore, the interaction between β-catenin and β-TrCP protein increased with the loss of FAM83H and weakened with overexpression of FAM83H (Figure 9F). The interaction between β-catenin and USP47 protein was weakened with the loss of FAM83H and increased with overexpression of FAM83H (Figure 9F). Therefore, these findings suggest that both FAM83H and SCRIB stabilize β-catenin protein by inhibiting its proteasomal degradation *via* interaction with β-TrCP and USP47.

## DISCUSSION

In the analysis of expression of FAM83H and SCRIB in human gastric tissue samples, their expression levels were significantly higher in cancer tissue compared with both non-neoplastic and precancerous lesions. Furthermore, their expression levels were much higher in advanced gastric carcinomas, suggesting that they play a role in the progression of gastric carcinomas. Similarly, elevated expression of the FAM83H gene was present in cancers of breast, colon, liver, lung, pancreas, and stomach [12]. Moreover, in this study, the expression of FAM83H-N and SCRIB-N predicted shorter survival of gastric carcinoma patients. Especially, the combined expression patterns of FAM83H-N and SCRIB-N were strongly predictive of the survival of gastric carcinoma patients. Consistently, FAM83H was an indicator of poor prognosis of cancers of the liver [4], kidney [5], uterus [10, 12], and bone [8]. The expression of SCRIB mRNA was also higher in hepatocellular carcinomas compared with non-tumorous liver tissue [20]. Also, the higher expression of SCRIB mRNA was significantly associated with poor prognosis of hepatocellular carcinoma [20] and breast cancer patients [24]. However, controversially, FAM83H expression was low in brain tumors, and increased expression of FAM83H was associated with favorable prognosis of patients [12]. Therefore, the prognostic impact of the expression of FAM83H and SCRIB might differ according to cancer type. However, despite some controversies, our data indicate that the survival of gastric carcinoma patients is independently associated with the expression of FAM83H-N and SCRIB-N. Moreover, the co-positivity of FAM83H-N and SCRIB-N was also an independent indicator of poor prognosis. Similarly, higher expression of SCRIB was associated with poor prognosis of estrogen receptor-negative and vimentin-positive breast cancer subtypes [25] and clear cell renal cell carcinoma patients [5]. Altogether, our results suggest that the expression of FAM83H-N and SCRIB-N might be novel prognostic indicators of gastric cancer patients.

In the cBioPortal public database, there was a strong correlation between the expression of FAM83H mRNA and SCRIB mRNA in gastric cancers (Pearson’s correlation; 0.77, Spearman’s correlation; 0.81, http://www.cbioportal.org) [6, 7]. In agreement with data from the public database, FAM83H expression showed a significant association with SCRIB expression in human gastric carcinoma tissue samples. Our in-depth univariate and multivariate analyses further found that the FAM83H-N^+^/SCRIB-N^+^ subgroup of gastric carcinoma had the shortest survival. Moreover, FAM83H and SCRIB stimulated the proliferation and invasion of gastric cancer cells, and FAM83H overexpression-stimulated proliferation of gastric cancer cells was attenuated with a knock-down of SCRIB *in vitro* and *in vivo*. The expression of mRNA and protein of SCRIB was significantly altered according to FAM83H expression. However, the expression of FAM83H was not influenced by SCRIB alteration. These findings suggest that FAM83H and SCRIB are closely related to the progression of gastric carcinomas, and that there are tumorigenic roles of FAM83H in mediating SCRIB; furthermore, the expression of SCRIB was dependent on FAM83H. However, despite the close correlation between FAM83H and SCRIB, the knock-down of SCRIB could not completely attenuate the effect of FAM83H overexpression in Figure 8. Therefore, there might be another role of FAM83H that is unrelated to SCRIB in cancer progression. Therefore, further study is necessary to explore the exact mechanism of how FAM83H is involved in cancer progression in conjunction with SCRIB or independent of SCRIB.

Concerning the role of FAM83H in conjunction with SCRIB in tumorigenesis, the oncogene MYC might be closely associated. In our previous study, we demonstrated that FAM83H was an intermediate of the oncogene MYC on the MYC-mediated proliferation and invasiveness of liver cancer cells [4]. The expression of FAM83H was transcriptionally controlled by MYC in liver cancer cells [4]. Cytoplasmic SCRIB was also involved in MYC-mediated hepatic tumorigenesis [20]. Although the mechanism by which SCRIB is involved in MYC-mediated tumorigenesis is puzzling, SCRIB is involved in MYC-mediated mammary tumorigenesis. In a mammary tumorigenic model, loss of SCRIB promoted tumorigenesis by suppressing MYC-mediated apoptosis, which was associated with the deregulation of SCRIB that results in the loss of its cytoplasmic membrane expression [19]. In addition, although there are only two cases of neuroendocrine carcinoma, nuclear expression of FAM83H in neuroendocrine carcinoma might be related to the MYC-FAM83H relationship because it has been reported that MYC drives progression of neuroendocrine carcinoma [26]. Therefore, our findings that FAM83H is involved in the expression of SCRIB suggests that the MYC-FAM83H-SCRIB pathway is important in tumorigenesis in both liver and stomach.

In this study, the effect of FAM83H and SCRIB on the proliferation of gastric cancer cells was associated with the canonical Wnt/β-catenin pathway. The reduced expression of both FAM83H and SCRIB decreased the protein level of β-catenin and the expression of cyclin D1. When considering the important role of the Wnt/β-catenin pathway on cellular proliferation, FAM83H and SCRIB had an effect on cellular proliferation through the regulation of the Wnt/β-catenin pathway by stabilizing the β-catenin protein. In our results, FAM83H and SCRIB were shown to bind to β-catenin and prevent its proteasomal degradation. Consistently, the possibility of FAM83H-mediated stabilization of β-catenin has been suggested in colorectal cancers [27] and osteosarcomas [8]. Furthermore, in this study, during the search for the roles and relationship between FAM83H and SCRIB in gastric carcinomas, we found that both FAM83H and SCRIB form a complex with β-catenin and are involved in post-translational stabilization from proteasomal degradation. As we have shown in Figure 9C, the expression of FAM83H and SCRIB are involved in nuclear localization of β-catenin. Therefore, nuclear expression patterns of FAM83H, SCRIB, and β-catenin and their interactions might be important in the progression of gastric cancers, and their nuclear expression may be of prognostic significance. The regulation of cytoplasmic and nuclear localization of β-catenin by escaping ubiquitination-mediated degradation is a classical model that explains how cancer develops in situations where the β-catenin degradation complex does not function due to inactivation or mutation of the degradation complex components, such as in the APC gene. Alternatively, the molecule might prevent degradation of β-catenin protein and might be involved in β-catenin activation-mediated proliferation of cells. In this context, our results present a model of β-catenin stabilization where β-catenin is protected from proteasomal degradation by forming a complex with FAM83H and SCRIB. Supportively, the interaction between β-catenin and β-TrCP protein was weakened by FAM83H and SCRIB, and the interaction between β-catenin and deubiquitinase USP47 protein was increased by FAM83H and SCRIB. Therefore, FAM83H and SCRIB stabilize β-catenin protein by protecting it from proteasomal degradation *via* interaction with β-TrCP and USP47. However, despite direct interaction between SCRIB and β-catenin, the localization of β-catenin was not affected by SCRIB [23]. Moreover, there is a controversial report that SCRIB is a negative regulator of Wnt/β-catenin signaling in a epithelial cell polarity model [28]. Therefore, further study is needed to explore the general or specific roles of FAM83H and SCRIB in the regulation of Wnt/β-catenin pathway during tumorigenesis.

In gastric cancer cells, FAM83H and SCRIB stimulated the invasiveness of cancer cells and FAM83H induced pulmonary metastasis in mice transplanted cancer cells with FAM83H overexpression. Acquiring invasive and metastatic potential is important in the progression of cancers, and EMT is an important phenotype of advanced carcinomas [18, 29, 30]. The hallmark of EMT is the deregulation of adhesion molecules with the loss of E-cadherin [14, 29, 31, 32]. FAM83H and SCRIB are also main components of cell adhesion and have roles in maintaining cytoskeletal structures [15, 27], and deregulated mislocalization of FAM83H and SCRIB are suggested to be involved in tumorigenesis [8, 19, 20, 24, 33]. Therefore, cytoplasmic and nuclear localization of FAM83H and SCRIB might be important in cancer progression *via* a regulation of EMT that accompanies disruption of cellular adhesion. Supportively, the cytoplasmic expression of SCRIB is inversely correlated with the membranous expression of E-cadherin [20]. In addition, as the degraded membranous components of SCRIB and E-cadherin, the levels of serum-soluble SCRIB and E-cadherin were higher in endometrial cancer patients compared with healthy volunteers [34]. Our results also showed that both FAM83H and SCRIB affected the expression of molecules closely associated with the EMT such as E-cadherin, N-cadherin, TGF-β1, snail, vimentin, MMP2, and MMP9. Moreover, our results indicate nuclear expression of FAM83H and SCRIB as independent indicators of poor prognosis of gastric carcinoma patients. In contrast to membranous SCRIB, cytoplasmic SCRIB stimulated the development and progression of tumors. Induced expression of cytoplasmic SCRIB *via* transfecting with mutant SCRIB promoted hepatic and mammary tumorigenesis by activating PI3K-Akt signaling [20, 24]. In the liver, cytoplasmic localization of SCRIB was higher in hepatocellular carcinoma compared with non-tumorous liver tissue, and cytoplasmic SCRIB induced expression of EMT genes [20]. In addition, EMT is another consequence of the activation of the Wnt/β-catenin pathway [29, 31, 35]. When considering the role of FAM83H and SCRIB in the stabilization of β-catenin, it might be suggested that cytoplasmic FAM83H and SCRIB form a complex with β-catenin in the cytoplasm and facilitate translocation to nuclei and activate the downstream signaling of the β-catenin pathway. In this manner, FAM83H- and SCRIB-mediated stabilization of β-catenin might activate cancer progression by regulating EMT. Therefore, when considering the importance of the loss of E-cadherin and activation of Wnt/β-catenin pathway in EMT, our results suggest that FAM83H-SCRIB-β-catenin-related alteration of the EMT pathway might be significantly involved in the progression of gastric cancer.

In conclusion, we demonstrate that FAM83H and SCRIB cooperatively activate the progression of gastric carcinoma by stabilizing β-catenin. Moreover, individual and co-expression patterns of the nuclear FAM83H and SCRIB might be used as novel prognostic markers of gastric cancers. Therefore, the FAM83H-SCRIB-β-catenin pathway might be a novel therapeutic target for the poor prognostic subgroups of gastric cancer, which express high levels of FAM83H and SCRIB.

## MATERIALS AND METHODS

### Human tissue samples and gastric carcinoma patients

To investigate the clinicopathological significance of the expression of FAM83H and SCRIB in human gastric carcinoma, this study evaluated 200 gastric carcinomas from patients operated on between January 1997 and December 2005 at Jeonbuk National University Hospital. In addition, tissue samples from 20 cases of non-neoplastic gastric mucosa unrelated with gastric cancer, and 40 cases of gastric dysplasia were included in this study. At first evaluation, the cases were staged according to the 6^th^ edition of the American Joint Committee Cancer Staging System [36], and 50 cases were selected from stage IV patients. Thereafter, 50 cases each were selected from stage I, II, and III gastric carcinomas and matched according gender, age (±2 y), and calendar year of operation (±2 y). The 200 cases of gastric carcinomas were classified according to WHO classification [22] and re-staged according to the 8^th^ edition of the American Joint Committee Cancer Staging System [37]. Information regarding clinicopathological factors was obtained through a review of the medical records and histologic reports. This study obtained institutional review board approval from Chonbuk National University Hospital (IRB number, CUH 2016-07-023-001) and was performed according to the Declaration of Helsinki. Based on the retrospective and anonymous character of the study the approval contained a waiver for written informed consent.

### Immunohistochemical staining of tissue samples and scoring

The expression of FAM83H, SCRIB, and β-catenin in human tissue samples were evaluated by immunohistochemical staining of tissue microarray sections. The tissue microarray was constructed from one 3.0 mm core area composed primarily of intact cells without degeneration. The tissue sections from the tissue microarray blocks were boiled with a microwave oven in pH 6.0 antigen retrieval solution (DAKO, Glostrup, Denmark) for 20 minutes. Immunohistochemical staining was performed with primary antibodies for the FAM83H (1:100, Bethyl Laboratories, Montgomery, TX), SCRIB (1:50, Santa Cruz Biotechnology, Santa Cruz, CA), and β-catenin (1:100, BD Biosciences, San Jose, CA). Immunohistochemical staining for FAM83H, SCRIB, and β-catenin were evaluated separately for nuclear and cytoplasmic expression by two pathologists (KYJ and KMK) with consensus. The staining intensity was scored as negative (score 0), weak (score 1), intermediate (score 2), and strong (score 3) staining and the staining area was scored as no staining (score 0), ~1% (score 1), 2~10% (score 2), 11~33% (score 3), 34~66% (score 4), and 67~100% (score 5) staining area [35, 38–40]. Thereafter, the sum of the staining intensity score and the staining area score was used as the immunohistochemical staining score ranging from zero to eight.

### Human gastric carcinoma cell lines and transfection

Two human gastric cancer cell lines, MKN-45 (wild type for FAM83H, SCRIB, CTNNB1, APC, GSK3B, AXIN1, and BTRC) and NCI-N87 (wild type for FAM83H, SCRIB, CTNNB1, APC, GSK3B, AXIN1, and BTRC) were purchased from the Korean Cell Line Bank (KCLB, Seoul, Republic of Korea). The indicated cell lines were grown in RPMI-1640 medium supplemented with 10% fetal bovine serum (Gibco BRL, Gaithersburg, MD) and streptomycin and penicillin (100 U/ml). The knock-down of FAM83H was induced with shRNA for FAM83H (GenePharma, Shanghai, China). The FAM83H duplex had the sense and antisense sequences 5′-CACCGCTCATCTTC AGCACGTCACATTCAAGAGATGTGACGTGCTGAAGATGAGCTTTTTTG-3′ and 5′-GATCCAAA AAAGCTCATCTTCAGCACGTCACATCTCTTGAATGTGACGTGCTGAAGATGAGC-3′, respectively. To induce knock-down of SCRIB, we used hSCRIB CRISPR/Cas9 KO plasmid (Catalog #; sc-400384, Santa Cruz Biotechnology, Santa Cruz, CA). The overexpression vector for SCRIB (Catalog #; EX-Z2845-M03, accession #; NM_002467) and FAM83H (Catalog #; EX-Y4473-M03, accession #; NM_198488) were purchased from GeneCopoeia (Rockville, MD). Lipofectamine^®^ 2000 DNA transfection reagent (Thermo Fisher Scientific, Waltham, MA) was used for transfection.

### Cell proliferation assay

Cell proliferation was evaluated by counting cells, a 3-(4,5-dimethylthiazol-2-yl)-2,5-diphenyltetrazolium bromide (MTT) cell proliferation assay, and a colony-forming assay. Cell counts were performed by seeding 3x10^3^ cells in a 6-well plate and counting with a hemocytometer after 24, 48, and 72 hours. The MTT assay was performed by seeding 3x10^3^ cells per well in 96 well plates for 24, 48, and 72 hours. The colony-forming assay was performed by plating 1x10^3^ cells per well in 6-well culture dishes in triplicates and allowing them to grow and form colonies for ten days. The culture plates were fixed with cold methanol and stained with 0.01% crystal violet for 1 hour; then, the number of colonies was counted with GeneTools analysis software (Syngene Imaging system, Frederick, MD).

### *In vitro* trans-chamber invasion and migration assay

To evaluate migration- and invasion-activity of cells *in vitro*, a 24-transwell migration or an invasion chamber (BD Biosciences, San Jose, CA) was used. The migration assay was performed by seeding 1x10^5^ cells in serum-free medium in the upper chamber with 8 μm-pore filters. The invasion assay was performed by seeding 2x10^5^ cells in serum-free medium in the upper 8μm-pore Matrigel Invasion Chamber (BD Biosciences, San Jose, CA). The bottom chamber consisted of 10% FBS containing RPMI-1640 for both migration and invasion assays. The migration and invasion chambers were incubated for 48 hours at 37 °C. The un-migrated and un-invaded cells on the upper surface of the chamber were removed using a cotton swab and the migrated and invaded cells on the underside of the insert were stained with DIFF-Quik staining solutions (Sysmex, Kobe, Japan). The migrated or invaded cells were counted in five microscopic fields (magnification x200) per well.

### Western blot, subcellular protein fractionation, and immunoprecipitation

To obtain protein, cells were lysed in ice-cold PRO-PREP Protein Extraction Solution (iNtRON Biotechnology, Seongnam, Korea). The primary antibodies used in this study were against FAM83H (Bethyl Laboratories, Montgomery, TX), SCRIB (Santa Cruz Biotechnology, Santa Cruz, CA), β-catenin (BD Biosciences, San Jose, CA), active (dephosphorylated) β-catenin (Millipore, Darmstadt, Germany), cyclin D1 (Cell Signaling Technology, Beverly, MA), E-cadherin (BD Biosciences, San Jose, CA), N-cadherin (Cell Signaling Technology, Beverly, MA), TGF-β1 (Cell Signaling Technology, Beverly, MA), snail (Abcam, Cambridge, UK), vimentin (Santa Cruz Biotechnology, Santa Cruz, CA), MMP2 (R&D Systems, Minneapolis, MN), MMP9 (Thermo Fisher Scientific, Fremont, CA), β-TrCP (Cell Signaling Technology, Beverly, MA), USP47 (Santa Cruz Biotechnology, Santa Cruz, CA), GSK3β (Cell Signaling Technology, Beverly, MA), phosphorylated GSK3β (Ser9) (Cell Signaling Technology, Beverly, MA), lamin B1 (Bioworld Technology, St. Louis Park, MN), GAPDH (Santa Cruz Biotechnology, Santa Cruz, CA), and actin (Santa Cruz Biotechnology, Santa Cruz, CA). Subcellular protein fractionation was performed using a Subcellular Protein Fractionation Kit (Thermo Scientific, Rockford, IL) according to the manufacturer’s protocol. The fractionated samples were resolved *via* SDS-PAGE and immunoblotted. Immunoprecipitation was performed using a Dynabeads-Protein A kit (Thermo Fisher Scientific, Rockford, IL) and 10-Tube Magnet (Invitrogen, Carlsbad, CA). The protein of interest was precipitated by pre-incubated 2 μg of primary antibodies with 1.5 mg of Dynabeads Protein A (Thermo Fisher Scientific, Rockford, IL) at room temperature. Then, 500 μg of diluted cell lysate was added and incubated with Dynabead-antibodies complex at 4°C overnight. The proteins in the antibody-bound complex were pelleted, washed three times with ice-cold protein extraction solution NP-40 (ELPiSBIO, Daejeon, Republic of Korea) and denatured with 2x Laemmli sample buffer at 95 °C for 10 minutes. The eluted proteins were detected by immunoblotting.

### Quantitative reverse-transcription polymerase chain reaction

Total RNA was obtained with an RNeasy Mini Kit (Qiagen Sciences, Valencia, CA) and reverse transcription was performed on 1.5 μg RNA with TaqMan Reverse Transcription Reagents (Applied Biosystems, Foster City, CA). A quantitative reverse-transcription polymerase chain reaction was performed with Applied Biosystems Prism 7900HT sequence Detection System and SYBR Green PCR Master Mix (Applied Biosystems, Foster City, CA). All experiments were carried out in triplicate, and the results were normalized to the expression of glyceraldehyde-3-phosphate dehydrogenase reference housekeeping gene. The primer sequences for the polymerase chain reaction are listed in Table 4.

**Table 4:** Primer sequences used for quantitative real-time polymerase chain reaction.

**Gene**	**Primer sequence**		**Product size**	**Accession number**
*FAM83H*	forward	CATGGTCCAGACAACCTGTG	214	NM_198488.3
	reverse	GCTGGATACCAGGAGGACAA		
*SCRIB*	forward	GGGACGACGAGGGCATATTC	207	NM_015356.5
	reverse	CGTTCTCAGGCTCCACCATGC		
*CTNNB1* (β-catenin)	forward	AAAATGGCAGTGCGTTTAG	100	NM_001904.3
	reverse	TTTGAAGGCAGTCTGTCGTA		
*CCND1* (Cyclin D1)	forward	GAGGAAGAGGAGGAGGAGGA	236	NM_053056.2
	reverse	GAGATGGAAGGGGGAAAGAG		
*E-cadherin*	forward	CCCGGGACAACGTTTATTAC	72	NM_004360.3
	reverse	ACTTCCCCTTCCTCAGTGAT		
*N-cadherin*	forward	ACAGTGGCCACCTACAAAGG	201	NM_001792.4
	reverse	CCGAGATGGGGTTGATAATG		
*TGF-β1*	forward	CCCACAACGAAATCTATGACAA	246	NM_000660.7
	reverse	AAGATAACCACTCTGGCGAGTG		
*SNAL1* (Snail)	forward	ACCCCACATCCTTCTCACTG	217	NM_005985.3
	reverse	TACAAAAACCCACGCAGACA		
*Vimentin*	forward	GAGAACTTTGCCGTTGAAGC	170	NM_003380.3
	reverse	TCCAGCAGCTTCCTGTAGGT		
*MMP2*	forward	CGGCCGCAGTGACGGAA	212	NM_004530.4
	reverse	CATCCTGGGACAGACGGAAGTTCTT	212	NM_004530.4
*MMP9*	forward	GACGCAGACATCGTCATCCA	200	NM_004994.2
	reverse	GCCGCGCCATCTGCGTTTCCAAA		
*GSK3B* (GSK3β)	forward	GCAGCAAGGTGACAACAGTG	979	NM_001146156.2
	reverse	AAGAGTGCAGGTGTGTCTCG		
*GAPDH*	forward	AACAGCGACACCCACTCCTC	258	NM_001256799.1
	reverse	GGAGGGGAGATTCAGTGTGGT		

Web link to accession numbers: https://www.ncbi.nlm.nih.gov/gene.

### Luciferase reporter assay

For cell-based luciferase reporter assay, gastric cancer NCI-N87 and MKN-45 cells were plated in 6-well plates at ~50% confluency. The cells were co-transfected with 100 ng/well TOPflash or FOPflash plasmid DNA and 2 ng/well pRL-TK *Renilla* luciferase plasmid DNA (Promega, Madison, WI) in addition to empty vectors, shRNA for FAM83H, hSCRIB CRISPR/Cas9 KO plasmid, overexpression vector for FAM83H, or overexpression vector for SCRIB by using Lipofectamine^®^ 2000 (Thermo Fisher Scientific, Waltham, MA). Twelve hours after transfection, cells were lysed with 1x Passive Lysis buffer using Dual-Glo Assay (Promega, Madison, WI). The firefly luciferase signals were monitored from 10 μl of each protein lysate by using the Dual-Luciferase Reporter Assay System (Promega, Madison, WI), and normalized to the signals obtained from positive controls of co-transfected Renilla luciferase expression. The assay was performed in quadruplicate and repeated three times.

### Tumorigenic assay

Eight-week-old male FoxnN.Cg/c nude mice (Orient Bio, Seongnam, Korea) were used to establish the xenografted gastric cancer model. The mice were randomly divided into four groups with five mice in each group. According to the experimental groups, NCI-N87 cells were transfected with empty vector, FAM83H-overexpressed vector, vectors to induce knockdown of SCRIB, or both of FAM83H-overexpression vector and vector for knockdown of SCRIB. After that, 2x10^6^ NCI-N87 cells were injected subcutaneously on the back. The size of the implanted tumor was measured every week, and the tumor volume was calculated by the equation: tumor volume = length x width x height x 0.52. At five weeks after tumor cell inoculation, the mice were euthanized after anesthetizing with sodium pentobarbital and evaluated for the tumors. All animal experiments were performed with the approval of the institutional animal care and use committee of Jeonbuk National University (approval number: CBNU 2018-033).

### Ubiquitin proteasomal degradation and ubiquitination analysis

MKN-45 gastric cancer cells were transfected with empty vector, shRNA for FAM83H, or hSCRIB CRISPR/Cas9 KO plasmid. After 24 hours post-transfection, the cells were treated with 30 μM cycloheximide (CHX; Sigma, St. Louis, MO) or 30 μM MG132 (Sigma, St. Louis, MO) for 30 minutes to two hours. Western blotting for β-catenin (BD Biosciences, San Jose, CA) and actin (Santa Cruz Biotechnology, Santa Cruz, CA) was performed on whole protein lysates. In addition, to evaluate the ubiquitination of β-catenin with knock-down of FAM83H or SCRIB, western blotting with anti-ubiquitin antibody after immunoprecipitation with anti-β-catenin antibody was performed. For this evaluation, MKN-45 cells were transfected with empty vector, shRNA for FAM83H, or hSCRIB CRISPR/Cas9 KO plasmid, and the cells were treated with 30 μM MG132 for two hours. The total cell lysates were immunoprecipitated with anti-β-catenin antibodies and blotted with anti-Ubiquitin antibody (Santa Cruz Biotechnology, Santa Cruz, CA).

### Statistical analysis

The positivity for the immunohistochemical expression of FAM83H, SCRIB, and β-catenin were determined by receiver operating characteristic curve analysis [31, 41, 42]. The cut-off points were determined at the points with the largest area under the curve to predict OS. Survival analysis was performed for OS and RFS of gastric carcinoma patients. An event in OS analysis was the death of the patients from gastric carcinoma, and the patients who were alive at last contact or died from other causes were treated as censored through June 2013. The relapse or death of patients from gastric carcinoma was an event of RFS analysis. The patients who were alive without relapse or died from other causes at last contact were treated as censored. The prognostic significance was analyzed with Cox proportional hazards regression analysis and Kaplan-Meier survival analysis. The relationship between factors was analyzed with Pearson’s chi-square test, Student’s *t*-test, and one-way ANOVA analysis. All experiments were done in triplicate, and the representative data are presented. All statistical analysis was performed using SPSS software (IBM, version 20.0, Armonk, NY). P values less than 0.05 were considered statistically significant.

## References

[1] Kim JW, Lee SK, Lee ZH, Park JC, Lee KE, Lee MH, Park JT, Seo BM, Hu JC, Simmer JP. FAM83H mutations in families with autosomal-dominant hypocalcified amelogenesis imperfecta. Am J Hum Genet. 2008; 82:489–94. 10.1016/j.ajhg.2007.09.020 18252228 PMC2427219

[2] Urzúa B, Martínez C, Ortega-Pinto A, Adorno D, Morales-Bozo I, Riadi G, Jara L, Plaza A, Lefimil C, Lozano C, Reyes M. Novel missense mutation of the FAM83H gene causes retention of amelogenin and a mild clinical phenotype of hypocalcified enamel. Arch Oral Biol. 2015; 60:1356–67. 10.1016/j.archoralbio.2015.06.016 26142250

[3] Nasseri S, Nikkho B, Parsa S, Ebadifar A, Soleimani F, Rahimi K, Vahabzadeh Z, Khadem-Erfan MB, Rostamzadeh J, Baban B, Banafshi O, Assadollahi V, Mirzaie S, et al. Generation of Fam83h knockout mice by CRISPR/Cas9-mediated gene engineering. J Cell Biochem. 2019. [Epub ahead of print]. 10.1002/jcb.28381 30714208

[4] Kim KM, Park SH, Bae JS, Noh SJ, Tao GZ, Kim JR, Kwon KS, Park HS, Park BH, Lee H, Chung MJ, Moon WS, Sylvester KG, Jang KY. FAM83H is involved in the progression of hepatocellular carcinoma and is regulated by MYC. Sci Rep. 2017; 7:3274. 10.1038/s41598-017-03639-3 28607447 PMC5468291

[5] Kim KM, Hussein UK, Bae JS, Park SH, Kwon KS, Ha SH, Park HS, Lee H, Chung MJ, Moon WS, Kang MJ, Jang KY. The expression patterns of FAM83H and PANX2 are associated with shorter survival of clear cell renal cell carcinoma patients. Front Oncol. 2019; 9:14. 10.3389/fonc.2019.00014 30723706 PMC6349742

[6] Gao J, Aksoy BA, Dogrusoz U, Dresdner G, Gross B, Sumer SO, Sun Y, Jacobsen A, Sinha R, Larsson E, Cerami E, Sander C, Schultz N. Integrative analysis of complex cancer genomics and clinical profiles using the cBioPortal. Sci Signal. 2013; 6:pl1. 10.1126/scisignal.2004088 23550210 PMC4160307

[7] Cerami E, Gao J, Dogrusoz U, Gross BE, Sumer SO, Aksoy BA, Jacobsen A, Byrne CJ, Heuer ML, Larsson E, Antipin Y, Reva B, Goldberg AP, et al. The cBio cancer genomics portal: an open platform for exploring multidimensional cancer genomics data. Cancer Discov. 2012; 2:401–04. 10.1158/2159-8290.CD-12-0095 22588877 PMC3956037

[8] Kim KM, Hussein UK, Park SH, Kang MA, Moon YJ, Zhang Z, Song Y, Park HS, Bae JS, Park BH, Ha SH, Moon WS, Kim JR, Jang KY. FAM83H is involved in stabilization of β-catenin and progression of osteosarcomas. J Exp Clin Cancer Res. 2019; 38:267. 10.1186/s13046-019-1274-0 31215499 PMC6582611

[9] Nalla AK, Williams TF, Collins CP, Rae DT, Trobridge GD. Lentiviral vector-mediated insertional mutagenesis screen identifies genes that influence androgen independent prostate cancer progression and predict clinical outcome. Mol Carcinog. 2016; 55:1761–71. 10.1002/mc.22425 26512949 PMC5393267

[10] Chen C, Li HF, Hu YJ, Jiang MJ, Liu QS, Zhou J. Family with sequence similarity 83 member H promotes the viability and metastasis of cervical cancer cells and indicates a poor prognosis. Yonsei Med J. 2019; 60:611–18. 10.3349/ymj.2019.60.7.611 31250574 PMC6597464

[11] Sasaroli D, Gimotty PA, Pathak HB, Hammond R, Kougioumtzidou E, Katsaros D, Buckanovich R, Devarajan K, Sandaltzopoulos R, Godwin AK, Scholler N, Coukos G. Novel surface targets and serum biomarkers from the ovarian cancer vasculature. Cancer Biol Ther. 2011; 12:169–80. 10.4161/cbt.12.3.16260 21617380 PMC3230481

[12] Snijders AM, Lee SY, Hang B, Hao W, Bissell MJ, Mao JH. FAM83 family oncogenes are broadly involved in human cancers: an integrative multi-omics approach. Mol Oncol. 2017; 11:167–79. 10.1002/1878-0261.12016 28078827 PMC5527452

[13] Elsum IA, Martin C, Humbert PO. Scribble regulates an EMT polarity pathway through modulation of MAPK-ERK signaling to mediate junction formation. J Cell Sci. 2013; 126:3990–99. 10.1242/jcs.129387 23813956

[14] Moreno-Bueno G, Portillo F, Cano A. Transcriptional regulation of cell polarity in EMT and cancer. Oncogene. 2008; 27:6958–69. 10.1038/onc.2008.346 19029937

[15] Martin-Belmonte F, Perez-Moreno M. Epithelial cell polarity, stem cells and cancer. Nat Rev Cancer. 2011; 12:23–38. 10.1038/nrc3169 22169974

[16] Huang L, Muthuswamy SK. Polarity protein alterations in carcinoma: a focus on emerging roles for polarity regulators. Curr Opin Genet Dev. 2010; 20:41–50. 10.1016/j.gde.2009.12.001 20093003 PMC3015045

[17] Navarro C, Nola S, Audebert S, Santoni MJ, Arsanto JP, Ginestier C, Marchetto S, Jacquemier J, Isnardon D, Le Bivic A, Birnbaum D, Borg JP. Junctional recruitment of mammalian scribble relies on e-cadherin engagement. Oncogene. 2005; 24:4330–39. 10.1038/sj.onc.1208632 15806148

[18] Thiery JP, Acloque H, Huang RY, Nieto MA. Epithelial-mesenchymal transitions in development and disease. Cell. 2009; 139:871–90. 10.1016/j.cell.2009.11.007 19945376

[19] Zhan L, Rosenberg A, Bergami KC, Yu M, Xuan Z, Jaffe AB, Allred C, Muthuswamy SK. Deregulation of scribble promotes mammary tumorigenesis and reveals a role for cell polarity in carcinoma. Cell. 2008; 135:865–78. 10.1016/j.cell.2008.09.045 19041750 PMC3015046

[20] Wan S, Meyer AS, Weiler SM, Rupp C, Tóth M, Sticht C, Singer S, Thomann S, Roessler S, Schorpp-Kistner M, Schmitt J, Gretz N, Angel P, et al. Cytoplasmic localization of the cell polarity factor scribble supports liver tumor formation and tumor cell invasiveness. Hepatology. 2018; 67:1842–56. 10.1002/hep.29669 29152770

[21] Bray F, Ferlay J, Soerjomataram I, Siegel RL, Torre LA, Jemal A. Global cancer statistics 2018: GLOBOCAN estimates of incidence and mortality worldwide for 36 cancers in 185 countries. CA Cancer J Clin. 2018; 68:394–424. 10.3322/caac.21492 30207593

[22] WHO Classification of Tumours Editorial Board. Digestive system tumours, International Agency for Research on Cancer. WHO classification of tumours digestive system tumours. Lyon: International Agency for Research on Cancer 2019.

[23] Sun Y, Aiga M, Yoshida E, Humbert PO, Bamji SX. Scribble interacts with beta-catenin to localize synaptic vesicles to synapses. Mol Biol Cell. 2009; 20:3390–400. 10.1091/mbc.e08-12-1172 19458197 PMC2710836

[24] Feigin ME, Akshinthala SD, Araki K, Rosenberg AZ, Muthuswamy LB, Martin B, Lehmann BD, Berman HK, Pietenpol JA, Cardiff RD, Muthuswamy SK. Mislocalization of the cell polarity protein scribble promotes mammary tumorigenesis and is associated with basal breast cancer. Cancer Res. 2014; 74:3180–94. 10.1158/0008-5472.CAN-13-3415 24662921 PMC4096808

[25] Sakakibara J, Sakakibara M, Shiina N, Fujimori T, Okubo Y, Fujisaki K, Nagashima T, Sangai T, Nakatani Y, Miyazaki M. Expression of cell polarity protein scribble differently affects prognosis in primary tumor and lymph node metastasis of breast cancer patients. Breast Cancer. 2017; 24:393–99. 10.1007/s12282-016-0715-2 27562784

[26] Mollaoglu G, Guthrie MR, Böhm S, Brägelmann J, Can I, Ballieu PM, Marx A, George J, Heinen C, Chalishazar MD, Cheng H, Ireland AS, Denning KE, et al. MYC drives progression of small cell lung cancer to a variant neuroendocrine subtype with vulnerability to aurora kinase inhibition. Cancer Cell. 2017; 31:270–85. 10.1016/j.ccell.2016.12.005 28089889 PMC5310991

[27] Kuga T, Kume H, Kawasaki N, Sato M, Adachi J, Shiromizu T, Hoshino I, Nishimori T, Matsubara H, Tomonaga T. A novel mechanism of keratin cytoskeleton organization through casein kinase iα and FAM83H in colorectal cancer. J Cell Sci. 2013; 126:4721–31. 10.1242/jcs.129684 23902688

[28] Daulat AM, Wagner MS, Walton A, Baudelet E, Audebert S, Camoin L, Borg JP. The tumor suppressor SCRIB is a negative modulator of the Wnt/β-catenin signaling pathway. Proteomics. 2019; 19:e1800487. 10.1002/pmic.201800487 31513346

[29] Kalluri R, Weinberg RA. The basics of epithelial-mesenchymal transition. J Clin Invest. 2009; 119:1420–28. 10.1172/JCI39104 19487818 PMC2689101

[30] Mendez MG, Kojima S, Goldman RD. Vimentin induces changes in cell shape, motility, and adhesion during the epithelial to mesenchymal transition. FASEB J. 2010; 24:1838–51. 10.1096/fj.09-151639 20097873 PMC2874471

[31] Bae JS, Park JY, Park SH, Ha SH, An AR, Noh SJ, Kwon KS, Jung SH, Park HS, Kang MJ, Jang KY. Expression of ANO1/DOG1 is associated with shorter survival and progression of breast carcinomas. Oncotarget. 2017; 9:607–21. 10.18632/oncotarget.23078 29416639 PMC5787493

[32] Kamikihara T, Ishigami S, Arigami T, Matsumoto M, Okumura H, Uchikado Y, Kita Y, Kurahara H, Kijima Y, Ueno S, Natsugoe S. Clinical implications of n-cadherin expression in gastric cancer. Pathol Int. 2012; 62:161–66. 10.1111/j.1440-1827.2011.02774.x 22360503

[33] Kuga T, Kume H, Adachi J, Kawasaki N, Shimizu M, Hoshino I, Matsubara H, Saito Y, Nakayama Y, Tomonaga T. Casein kinase 1 is recruited to nuclear speckles by FAM83H and SON. Sci Rep. 2016; 6:34472. 10.1038/srep34472 27681590 PMC5041083

[34] Ouyang Z, Chen M, Sun J, Zhai J. Expression and role of hScrib in endometrium, endometriosis, and endometrial adenocarcinoma. Medicine (Baltimore). 2019; 98:e14076. 10.1097/MD.0000000000014076 30702562 PMC6380690

[35] Bae JS, Noh SJ, Kim KM, Park SH, Hussein UK, Park HS, Park BH, Ha SH, Lee H, Chung MJ, Moon WS, Cho DH, Jang KY. SIRT6 is involved in the progression of ovarian carcinomas via β-catenin-mediated epithelial to mesenchymal transition. Front Oncol. 2018; 8:538. 10.3389/fonc.2018.00538 30524965 PMC6256124

[36] Greene FL, American Joint Committee on Cancer. American Cancer Society. AJCC cancer staging Handbook : from the AJCC cancer staging manual. New York: Springer 2002 10.1007/978-1-4757-3656-4

[37] Amin MB, American Joint Committee on Cancer., American Cancer Society. AJCC cancer staging manual. Chicago IL: American Joint Committee on Cancer, Springer 2017 10.1007/978-3-319-40618-3

[38] Allred DC, Harvey JM, Berardo M, Clark GM. Prognostic and predictive factors in breast cancer by immunohistochemical analysis. Mod Pathol. 1998; 11:155–68. 9504686

[39] Park HJ, Bae JS, Kim KM, Moon YJ, Park SH, Ha SH, Hussein UK, Zhang Z, Park HS, Park BH, Moon WS, Kim JR, Jang KY. The PARP inhibitor olaparib potentiates the effect of the DNA damaging agent doxorubicin in osteosarcoma. J Exp Clin Cancer Res. 2018; 37:107. 10.1186/s13046-018-0772-9 29784019 PMC5963190

[40] Kang MA, Lee J, Ha SH, Lee CM, Kim KM, Jang KY, Park SH. Interleukin4Rα (IL4Rα) and IL13Rα1 are associated with the progress of renal cell carcinoma through janus kinase 2 (JAK2)/forkhead box O3 (FOXO3) pathways. Cancers (Basel). 2019; 11:1394. 10.3390/cancers11091394 31540495 PMC6770213

[41] Bae JS, Park SH, Jamiyandorj U, Kim KM, Noh SJ, Kim JR, Park HJ, Kwon KS, Jung SH, Park HS, Park BH, Lee H, Moon WS, et al. CK2α/CSNK2A1 phosphorylates SIRT6 and is involved in the progression of breast carcinoma and predicts shorter survival of diagnosed patients. Am J Pathol. 2016; 186:3297–315. 10.1016/j.ajpath.2016.08.007 27746184

[42] Noh SJ, Kim KM, Jang KY. Individual and co-expression patterns of nerve growth factor and heme oxygenase-1 predict shorter survival of gastric carcinoma patients. Diagn Pathol. 2017; 12:48. 10.1186/s13000-017-0644-1 28679437 PMC5498870

